# Inhibition of Metastasis by Polypyridyl Ru(II) Complexes through Modification of Cancer Cell Adhesion – *In Vitro* Functional and Molecular Studies

**DOI:** 10.1021/acs.jmedchem.2c00580

**Published:** 2022-07-27

**Authors:** Ilona Gurgul, Ewelina Janczy-Cempa, Olga Mazuryk, Małgorzata Lekka, Michał Łomzik, Franck Suzenet, Philippe C. Gros, Małgorzata Brindell

**Affiliations:** † Faculty of Chemistry, Jagiellonian University in Krakow, Gronostajowa 2, 30-387Krakow, Poland; ‡ Department of Biophysical Microstructures, Institute of Nuclear Physics, Polish Academy of Sciences, PL-31342Krakow, Poland; § Department of Organic Chemistry, Faculty of Chemistry, University of Łódź, ul. Tamka 12, 91-403Łódź, Poland; ∥ Institute of Organic and Analytical Chemistry, University of Orléans, UMR-CNRS 7311, rue de Chartres, BP 6759, 45067Orléans Cedex 2, France; ⊥ Université de Lorraine, CNRS, L2CM, F-54000Nancy, France

## Abstract

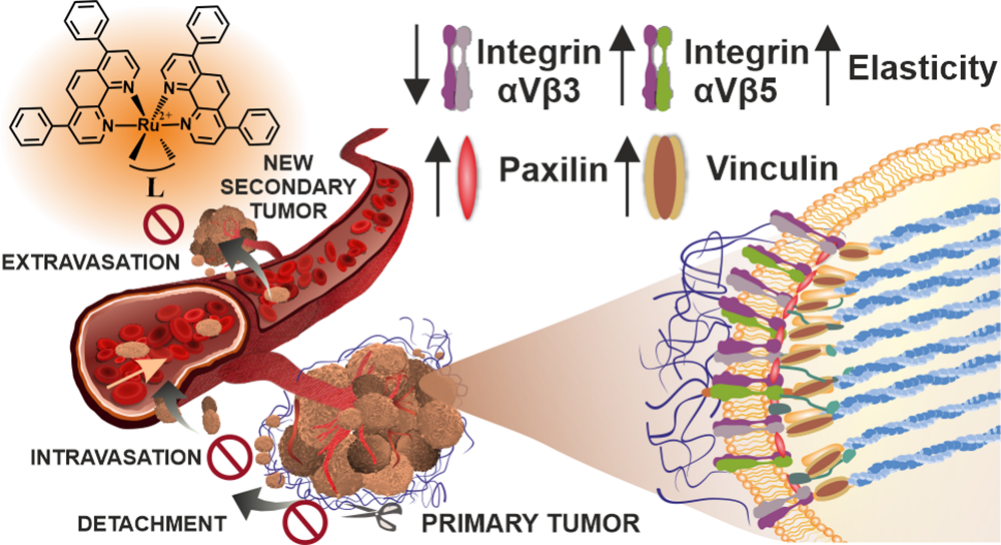

The effect of polypyridyl Ru(II) complexes on the ability of cancer cells to migrate and invade, two features important in the formation of metastases, is evaluated. *In vitro* studies are carried out on breast cancer cell lines, MDA-MB-231 and MCF-7, as well as melanoma cell lines A2058 and A375. Three Ru(II) complexes comprising two 4,7-diphenyl-1,10-phenanthroline (dip) ligands and as a third ligand 2,2′-bipyridine (bpy), or its derivative with either 4-[3-(2-nitro-1H-imidazol-1-yl)propyl] (bpy-NitroIm), or 5-(4-{4′-methyl-[2,2′-bipyridine]-4-yl}but-1-yn-1-yl)pyridine-2-carbaldehyde semicarbazone (bpy-SC) moiety attached are examined. The low sub-toxic doses of the studied compounds greatly affected the cancer cells by inhibiting cell detachment, migration, invasion, transmigration, and re-adhesion, as well as increasing cell elasticity. The molecular studies revealed that the Ru(II) polypyridyl complexes impact the activity of the selected integrins and upregulate the expression of focal adhesion components such as vinculin and paxillin, leading to an increased number of focal adhesion contacts.

## 1 Introduction

Undoubtedly, chemotherapy is a powerful weapon in the fight against cancer. Despite the increasing amount of data on the effects of chemotherapeutic agents, which were drawn not only from *in vivo* research but also from clinical trials and therapy, there are still some problems to be solved. An important question that researchers have recently raised is the link between chemotherapy and the promotion of cancer metastasis.^1^ However, this topic is little investigated and there are only a few reports that address this problem in *in vivo* or clinical studies. For example, *in vivo* studies on human breast xenograft models in mice showed that basic anticancer drugs in the adjuvant and neoadjuvant treatment of cancer can promote the formation of distant metastases through increased intravasation.^2^ The clinical results addressed to this issue are not clear.^3^ There is no direct evidence (data) that pro-metastatic changes in ER+ breast cancers result from the use of neoadjuvant chemotherapy.^4^ Importantly, a benefit in the application of neoadjuvant chemotherapy manifested in improved survival in complete pathological response was evident. Therefore, one must be very careful in formulating conclusions from these studies and this issue requires further extensive research and clarification. Other clinical studies revealed the negative impact of tamoxifen on disease-free survival and metastasis-free survival of patients with ERα36+ breast cancer treated with this drug after surgery.^5^ It was proposed that tamoxifen interacts directly with ERα36+ expressed by breast cancer cells, which in turn induces proliferation and metastasis of breast cancer. Generally, clinical studies are very challenging because they deal with a heterogeneous group of patients with diverse prognoses; therefore, large-scale studies considering various parameters are needed to establish if there is indeed a link between systemic therapies and metastasis. However, in recent years, there have been voices from oncologists and scientists that research on new drugs should also include studies to determine whether chemotherapy can promote metastases.^6^

Achieving the cytotoxic concentration of drugs reaching cancer cells is often prevented by their poor biodistribution resulting from deprived tumor vascularization and the occurrence of hypoxia. Therefore, typically maximum tolerated doses (MTD) are applied in the treatment, which leads to host toxicity and diverse side effects. However, recently, low-dose metronomic (LDM) chemotherapy was suggested as an alternative form of chemotherapy, particularly for those patients who may not be considered for MTD due to poor health conditions.^7^ In LDM chemotherapy, low doses of drugs are administered on a frequent or continuous schedule without extended interruptions. Although there are only a few available studies, some observations indicated that LDM chemotherapy might induce less metastasis-favorable changes in the tumor microenvironment than standard chemotherapy.^1b^ Clinical trials on its evaluation (*Effect of Low Dose Metronomic Chemotherapy in Metastatic Breast Cancer*, ClinicalTrials.gov Identifier: NCT04350021) are currently in progress. Therefore, for any new potential cytotoxic agent, it is important to know how non-toxic doses affect cancer cells and whether they can have any beneficial effects or on the contrary may increase the risk of developing distant metastasis.

Among the compounds tested as antimetastatic drugs, one of the ruthenium complexes, namely, imidazolium *trans*-[tetrachlorido(1H-imidazole)(*S*-dimethylsulfoxide)ruthenate(III)], known as NAMI-A, and its sodium salt precursor, (NAMI), deserves the attention of researchers.^8^ NAMI-A was the first and only metal complex that entered clinical trials as a nontoxic compound exhibiting antimetastatic properties in animal models. Their unique features arise from inhibiting the main stages of the dissemination process.^8c^ Among others, it significantly increased the adhesion strength of cells and reduced the rates of invasion and transmigration through endothelial cells. Furthermore, it inhibited the secretion of proteolytic enzymes such as MMP-2 and MMP-9, which play a significant role in cancer cell survival and expansion since they are involved in all stages of carcinogenesis. It modulated the tumor microenvironment by changing the activity and/or expression of various adhesion molecules and integrins. Despite the failure of the clinical investigation, research on NAMI-A development by Enzo Alessio, Gianni Sava, and Giovanni Mestroni became a milestone in the field of anticancer metal compounds, directing the attention of researchers to antimetastatic studies. Another group of compounds that exerted antimetastatic activity with low cytotoxic activity was a series of organometallic ruthenium(II)-arene complexes developed by Dyson et al. RAPTA-C, [Ru(*p*-cymene)Cl_2_(PTA)] (PTA - 1,3,5-triaza-7-phosphaadamantane), has been particularly well studied, and its antimetastatic activity was assigned to its binding to the histone proteins in nucleosome core particles by alternating chromatin compaction.^9^ Research has recently commenced on Ru polypyridyl complexes to test their potential to inhibit metastasis development, with promising results.^10^

**Figure 1:**
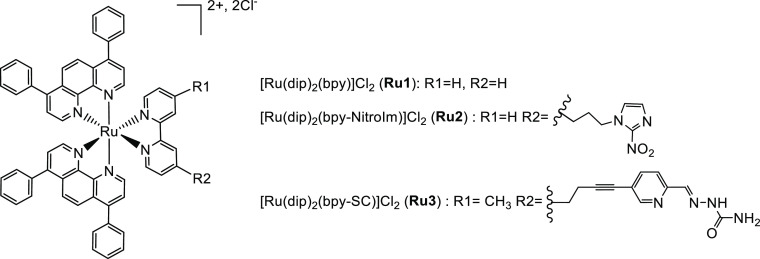
Studied ruthenium(II) complexes.

In this work, we address the issues mentioned above by examining the effect of the tested compounds in low sub-toxic doses on the properties of cancer cells, which are important for their ability to metastasize. Our previous studies on human lung adenocarcinoma A549 and human pancreatic carcinoma PANC-1 showed that **Ru1** and **Ru3** (the structures are depicted in Figure 1) substantially decreased cells’ susceptibility to detachment when cultured either on plastic or collagen-coated surfaces. On the other hand, cells pre-treated with the same Ru(II) complexes exhibited a lower ability to re-adhere to a substrate after detachment.^11^ A similar effect on breast cancer 4T1 cell adhesion was observed after **Ru2** treatment (the structure is depicted in Figure 1).^12^ Cell adhesion is a functional characteristic of cells that plays a crucial role in cancer progression and metastasis. Furthermore, we have shown that **Ru3** inhibited the released and membrane-bound metalloproteinases (MMPs) in A549 cells, while in model studies, both **Ru1** and **Ru3** directly inhibited MMP-2 and MMP-9 enzyme activities.^13^ Such preliminary findings encouraged us to select these complexes (**Ru1**–**Ru3**, see Figure 1) as good candidate compounds for testing their potency in the inhibition of metastasis by influencing cell detachment, migration, invasion, transmigration, and re-adhesion, as well as their elasticity. For *in vitro* studies, two breast cancer cell lines, MDA-MB-231 and MCF-7, and two melanoma cell lines, A2058 and A375, were chosen. MDA-MB-231, a triple-negative breast adenocarcinoma cell line, is highly invasive, aggressive, and poorly differentiated. These properties are due to the lack of expression of estrogen (ER) and progesterone (PR) receptors, as well as human epidermal growth factor receptor (HER2).^14^ The invasion of MDA-MB-231 cells was related to their ability for proteolytic degradation of the extracellular matrix (ECM). MCF-7 is an estrogen-responsive (ER-positive and PR-positive) breast adenocarcinoma cell line, which is generally considered to have low metastatic potential.^15^ A2058 is a highly invasive melanoma cell line characterized by high collagen type IV collagenase expression and low endogenous expression of Wnt5a. High levels of αv integrin expression, tissue inhibitor of metalloproteinase-2, and autocrine motility factor are responsible for its high metastatic potential.^16^ A375 is a human malignant melanoma cell line with low metastatic potency, characterized by the presence of adenosine receptors, responsible for modulating tumor processes.^17^ Studies for the melanoma cells were also carried out under hypoxia (low oxygen conditions) due to the multidirectional influence of hypoxia on the metastatic cascade resulting from reprogramming the cellular metabolism and signaling.^18^ Furthermore, to get insights into the molecular basis of the observed cellular functional changes induced by the Ru(II) polypyridyl complexes, the expression of focal adhesion components such as vinculin and paxillin and the resulting number of focal adhesion contacts as well as the cell’s mechanical properties were investigated.

## 2 Results and Discussion

### 2.1 Cytotoxicity and Uptake of Ru Complexes

The cytotoxic effect of **Ru1**, **Ru2**, and **Ru3** evaluated on two melanoma cell lines, A375 and A2058, two breast cancer cell lines, MCF-7 and MDA-MB-231, and non-cancerous immortalized keratinocytes, HaCat, is shown in Table 1. All three complexes displayed moderate to high cytotoxic effects depending on the studied cell line, and their cytotoxicity was much higher than that of the well-known cisplatin. An especially promising property is their particular cytotoxicity against triple-negative breast cancer MDA-MB-231 cells since cisplatin treatment of triple-negative breast cancer often results in the development of chemoresistance.^19^ Application of all compounds to cells grown under hypoxic conditions resulted in a decrease in their cytotoxic efficacy. Notably, for all three Ru complexes, the cytotoxicity against the human non-tumor HaCat cells was lower than that against cancer cells. At the same time, cisplatin remained at the same level. Among others, the Ru complexes were 5–20 times less cytotoxic against HaCat than against MDA-MB-231 cells.

**Table 1:** Cytotoxicity (IC_50_) of the Ru(II) Complexes and Cisplatin Evaluated for A375, A2058, MCF-7, MDA-MB-231, and HaCat Cells under Normoxia (21% O_2_) and Hypoxia (1% O_2_) Conditions

	IC_50_ (μM)			
cell line (conditions)	**Ru1**	**Ru2**	**Ru3**	cisplatin
A375 (normoxia)	9.7 ± 0.4	11.2 ± 0.9	15.0 ± 0.6	61 ± 5
A375 (hypoxia)	8.4 ± 0.2	20 ± 1	12 ± 2	145 ± 30
A2058 (normoxia)	4.9 ± 0.9	10.8 ± 0.8	4.7 ± 0.5	53 ± 9
A2058 (hypoxia)	6.7 ± 0.3	18 ± 3	15 ± 2	182 ± 44
MCF-7 (normoxia)	3.9 ± 0.6	13 ± 2	13.1 ± 0.3	54 ± 6
MDA-MB-231 (normoxia)	0.8 ± 0.6	3.8 ± 0.2	1.8 ± 0.3	82 ± 3
HaCat (normoxia)	22 ± 3	19 ± 4	27.7 ± 0.8	71 ± 14
HaCat (hypoxia)	14 ± 3	16 ± 2	18.6 ± 0.6	41 ± 2

**Figure 2:**
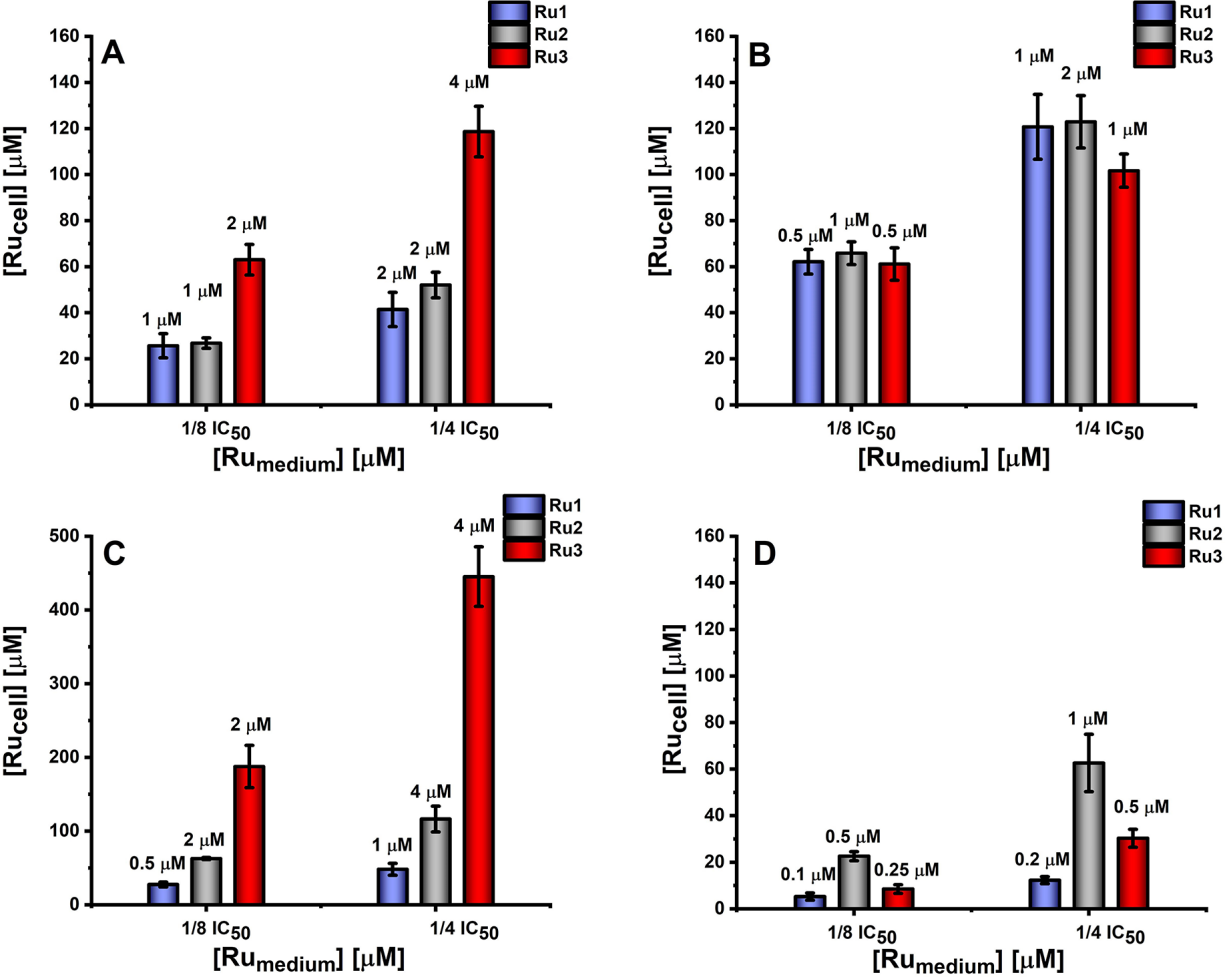
Ruthenium accumulation in (A) A375, (B) A2058, (C) MCF-7, and(D) MDA-MB-231 determined after 24 h of incubation with [Ru(dip)_2_(bpy)]Cl_2_ (**Ru1**, blue), [Ru(dip)_2_(bpy-NitroIm)]Cl_2_ (**Ru2**, gray), and [Ru(dip)_2_(bpy-SC)]Cl_2_ (**Ru3**, red) presented as concentration in a single cell (obtained from ICP-MS measurements). Concentrations for individual Ru(II) compounds applied as 1/4 or 1/8 of IC_50_ are given above each bar.

The accumulation of Ru ions in various cell lines was studied for all tested compounds at concentrations of 1/8 IC_50_ and 1/4 IC_50_ to check the amount of individual compounds needed to exert the same biological effect. The amounts of Ru ions accumulated in cells evaluated by ICP-MS and the concentrations used for incubation are shown in Figure 2. The three compounds accumulated readily in each type of cell used, as clearly revealed by an increase in the concentration of ruthenium accumulated in cells in relation to the concentration in the cell medium, 20 to even 120 times. Cellular uptake of complexes was dose-dependent and strongly dependent on the cell line (Figure 2). Interestingly, the highest cytotoxicity to MDA-MB-231 cells was not due to the high accumulation of compounds, but rather to the susceptibility of the cells to their activity. MCF-7 cells remained the most resistant, especially to **Ru3**, requiring a very high concentration of ca. 450 μM in the cell to obtain 1/4 IC_50_, while for the other cell lines, it was below 120 μM. The propensity to accumulate in cells is an important factor in cytotoxic activity, but the ability to reach and interact with the appropriate targets in the cell plays a key role.

### 2.2 Impact on Cancer Cell Adhesion and Re-adhesion Properties

**Figure 3:**
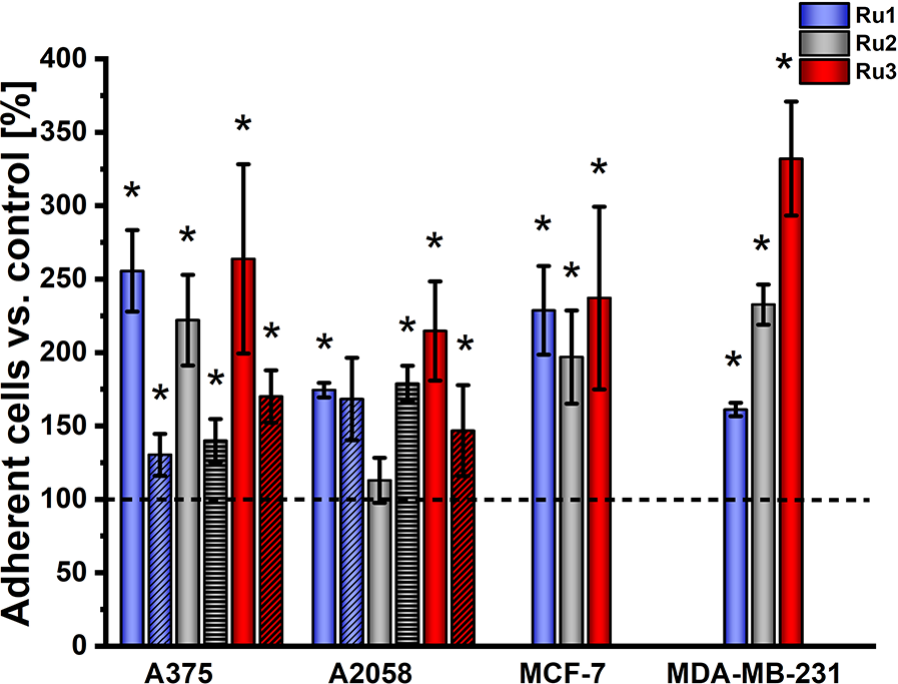
Cell adhesion was evaluated as a percentage of adherent cells that remained after controlled trypsin treatment. Cells were incubated with [Ru(dip)_2_(bpy)]Cl_2_ (**Ru1**,blue), [Ru(dip)_2_(bpy-NitroIm)]Cl_2_ (**Ru2**, gray), and [Ru(dip)_2_(bpy-SC)]Cl_2_ (**Ru3**, red) at a concentration equal to 1/8 of IC_50_ under normoxic (filled bar) and hypoxic (dashed bar) conditions. Untreated cells were used as a control (100%, dashed line). *p < 0.05.

The potential of Ru(II) compounds to increase cell adhesion properties was verified by applying the trypsin resistance assay.^10^ Cells, 24 h after the treatment with the tested compounds, were exposed to a diluted trypsin solution for a short time to minimize cell disruption, and the amount of remaining trypsin-resistant cells was quantified using a resazurin assay. The studies were carried out on cells with different invasiveness potentials, and the obtained results indicated that the treatment of highly metastatic A2058 cells with the Ru(II) compounds had a rather low effect on their adherence to plastic under both normoxic and hypoxic conditions (Figure 3). On the other hand, cells from the other lines exhibited even a doubled adherence under normoxic conditions (Figure 3). Treatment of highly metastatic MDA-MB-231 cells with **Ru3** resulted in a pronounced reinforcement of cell adhesion. The increase in the concentration of compounds from 1/8 of IC_50_ to 1/4 of IC_50_ had no evident impact on the observed effects (Figure S1). However, a decreased effect on adhesion was noted for A375 cells growth under hypoxia. Therefore, the observed effect strongly depends on the type of cell line, pointing to a large heterogeneity between cancer cells.

**Figure 4:**
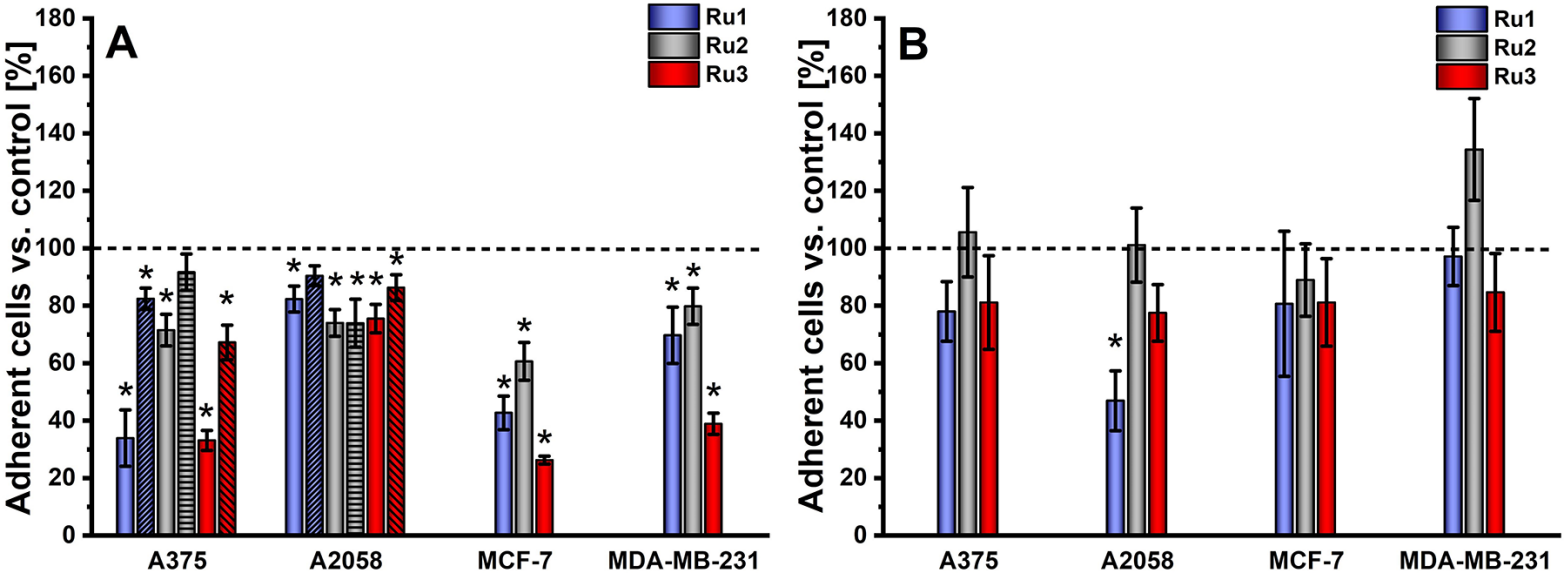
Cells’ ability to re-adhere to plastic (A) or monolayer of endothelial cells (B), measured after 24 h of incubation with [Ru(dip)_2_(bpy)]Cl_2_ (**Ru1**, blue), [Ru(dip)_2_(bpy-NitroIm)]Cl_2_ (**Ru2**, gray), and [Ru(dip)_2_(bpy-SC)]Cl_2_ (**Ru3**, red) under normoxic (filled bar) and hypoxic (dashed bar) conditions ([Ru] = 1/4 IC_50_). Untreated cells were used as a control (100%, dashed line). *p < 0.05.

In addition, the re-adhesion of cells treated with the Ru compounds was evaluated. The re-adhesion of cells to the substrate (other cells or extracellular matrix) is important for cell survival and proliferation.^21^ Cells treated with non-toxic doses of Ru complexes (1/4 IC_50_ and 1/8 IC_50_) were detached after 24 h of incubation and re-seeded into 96-well plates. The time necessary for tumor cell adhesion depends on the tumor type, but usually occurs within 10 to 30 min, so the chosen 1 h of incubation was enough to obtain reliable control. As shown in Figure 4A and Figure S2, the most significant inhibition of cell adhesion was observed with **Ru3** followed by **Ru1** in the three cell lines A375, MCF-7, and MDA-MB-231, while for A2058 cells, the effect was marginal. The induced decrease in re-adhesion was concentration-dependent, particularly for A375 cells under normoxic conditions (Figure S2). Hypoxic conditions made A375 cells less affected by the treatment with Ru(II) complexes, preserving their re-adhesion properties close to the control. The low impact of Ru compounds on the adhesion and re-adhesion properties of A375 cells under hypoxic conditions suggests that oxygen-deprived conditions induce phenotypic changes in cells, making them more resistant to molecular changes induced by the studied compounds.

Furthermore, the ability of cancer cells pre-treated with the Ru complexes to adhere to a monolayer of endothelial cells was assessed. This process is relevant for intravasation and extravasation, where the initial arrest and attachment of tumor cells to the vascular endothelium is a prerequisite. To distinguish cancer cells from endothelial cells, the former cells were fluorescently labeled. As shown in Figure 4B, the adherence to endothelial cells is only significantly influenced in the case of treatment of A2058 cells with **Ru1**.

Such an effect of Ru compounds on the adhesion properties of cancer cells, i.e., reinforcement of adhesion and inhibition of re-adhesion of cells to the plastic surface, may arise from the involvement of a different set of cell adhesion molecules (CAM) engaged in receptor-mediated cell de-adhesion from the surface compared to the re-adhesion process. In both cases, **Ru3** exhibited high activity in blocking these processes; however, its activity depends on the cell line. The obtained results confirm the existence of a clear heterogeneity between cell lines, further exacerbated by hypoxic conditions. At the molecular level, one of the possible targets for Ru complexes would be integrins that are involved in the direct cell–cell or cell–substrate (ECM) interactions responsible for adhesion. In addition, other components of focal adhesion can be involved in the detachment process. This issue is further discussed in detail below*.*

### 2.3 Impact on the Migration, Invasion, and Transmigration of Cancer Cells

**Figure 5:**
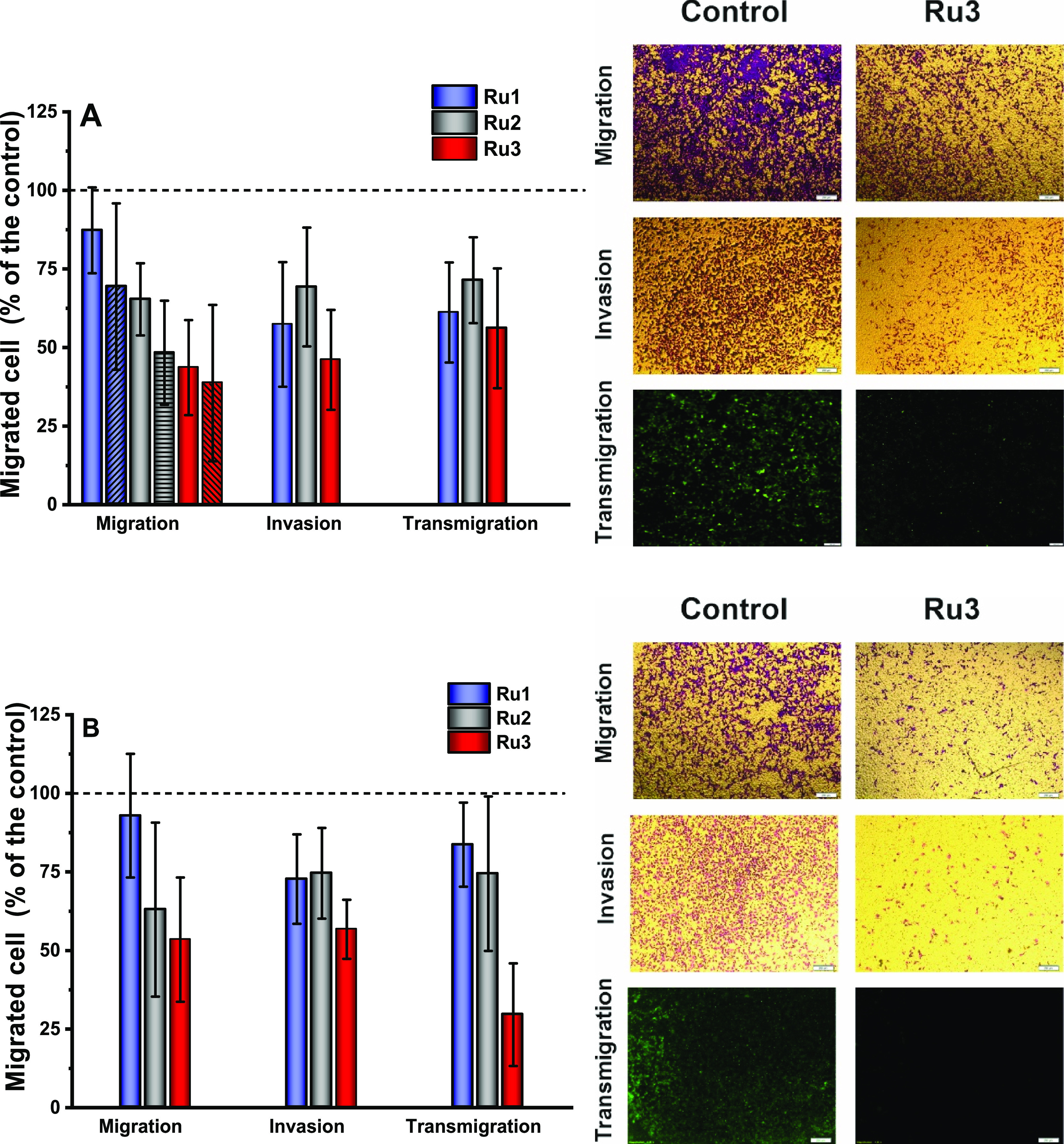
Effects of [Ru(dip)_2_(bpy)]Cl_2_ (**Ru1**, blue), [Ru(dip)_2_(bpy-NitroIm)]Cl_2_ (**Ru2**, gray) and [Ru(dip)_2_(bpy-SC)]Cl_2_ (**Ru3**, red) on migration, invasion, and transmigration of (A) A375 and (B) MDA-MB-231 cells under normoxic (filled bar) and hypoxic (dashed bar) conditions. ([Ru] = 1/8 IC_50_) Untreated cells were used as control (100%, dashed line).

Cell migration is an important feature of metastatic cells and is involved in many steps of the metastasis cascade. Cell migration is a complex process that, among others, involves a subtle balance of adhesion and detachment, cytoskeleton remodeling, and the generation of protrusive force and is strongly influenced/regulated by the extracellular matrix.^22^ The effect of the investigated Ru compounds on cell migration was studied using a trans-membrane migration assay (Transwell chamber).^10^ To evaluate the impact of the Ru(II) complexes on cell migration, cells were seeded in the upper chamber, and an appropriate concentration of Ru(II) compound was added in a medium supplemented with 1% FBS. Cell movement to a lower well containing medium with the same concentration of Ru(II) compound and 20% FBS separated from the upper chamber by a microporous membrane was quantified after 16 h by counting cells stained with crystal violet in the lower chamber. The chemotactic gradient created just by the addition of serum in the lower well was enough to promote migration of A375 and MDA-MB-231, while A2058 and MCF-7 had to be starved in serum-free medium for 24 h prior to the experiment. As shown in Figure 5 and Figures S3 and S4, all three studied compounds inhibited migration; however, only **Ru3** was able to suppress mobility above 50%. Hypoxic conditions tested for A375 cells had no significant effect on the inhibitory effect of Ru compounds. Another protocol involving preincubation of cells with the Ru(II) complexes for 24 h prior to seeding them in the upper chamber was applied to evaluate how changes in adhesion properties induced by Ru complexes influence cell mobility. Again, **Ru3** was also the most efficient inhibitor (Figures S5 and S6). Generally, the inhibition of cell mobility was slightly stronger than cells treated directly in the upper chamber, which may explain the hampered detachment of cells from the plastic surface in the case of direct incubation of cells with Ru complexes on the inserts.

The invasion was evaluated using a Transwell chamber, in which a microporous membrane was pre-coated with a Matrigel matrix. The invasion was assessed for A375 and MDA-MB-231 cells, which were characterized by high mobility. For both cell lines, **Ru3** was the strongest inhibitor of invasion, and the observed effect was concentration-dependent (Figure 5 and Figure S7). The invasion of MDA-MB-231 cells was less attenuated by all tested compounds compared to A375 cells. For a higher concentration (1/4 IC_50_), all three compounds suppressed invasion by more than 50% in A375 cells, while in MDA-MB-231, only **Ru3** was very efficient. This may be explained by a much higher invasion potency of MDA-MB-231 cells.^23^

Furthermore, the trans-endothelial migration through the monolayer of endothelial HMEC-1 cells was evaluated. To differentiate between cancer and endothelial cells, the former cells were fluorescently labeled. Treatment of cancer cells with the studied compounds led to a reduction in the total number of transmigrating cells at a level similar to that found for the Matrigel, and again, the **Ru3** compound exhibited the strongest inhibitory activity (Figure 5 and Figure S8). Crossing endothelial barriers is required for intra- and extravasation processes, and blocking these processes can help to inhibit the spread of cancer cells.

Mobility, invasion, and transmigration ability decreased significantly in studied cells treated with **Ru3** compared to non-treated control cells. In further studies, an attempt was made to identify molecular targets responsible for the observed effects induced in cells by the Ru compounds.

### 2.4 Alteration of Integrin Accessibility

**Figure 6:**
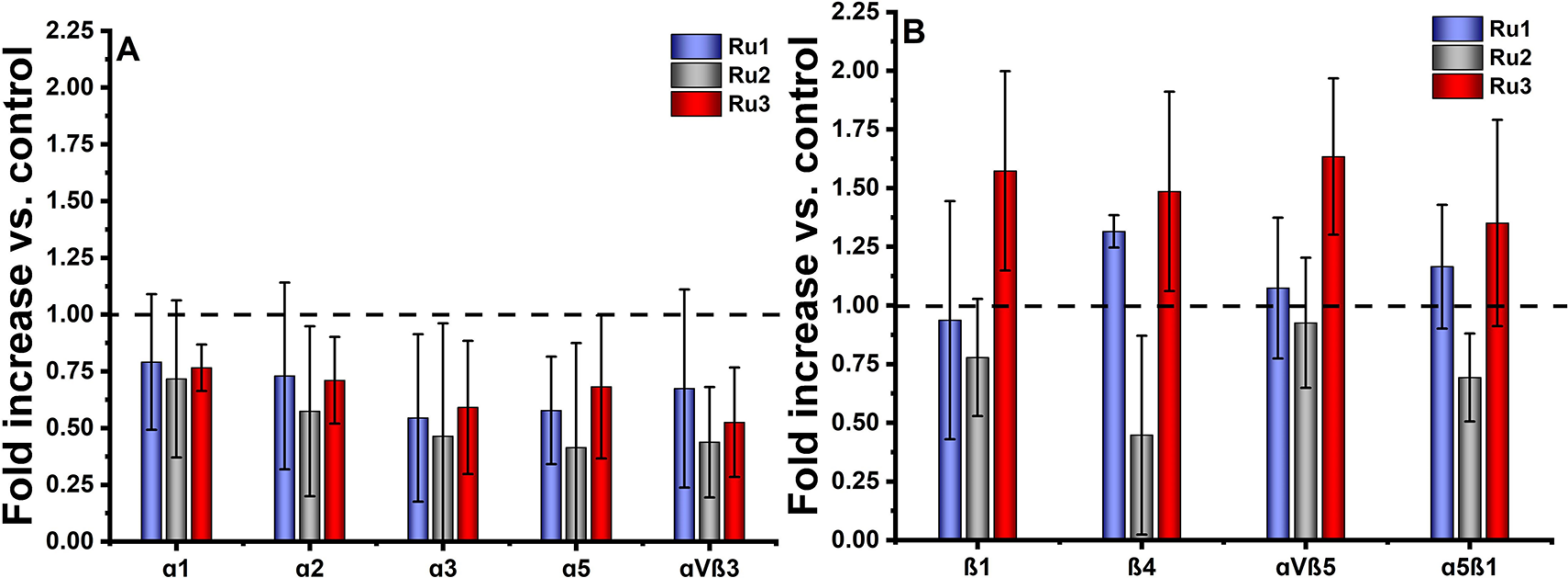
Protein expression/binding profile of α (A) and β (B) integrin subunits and heterodimers on MDA-MB-231 cells measured after 2 h of incubation with [Ru(dip)_2_(bpy)]Cl_2_ (**Ru1**, blue), [Ru(dip)_2_(bpy-NitroIm)]Cl_2_ (**Ru2**, gray), and [Ru(dip)_2_(bpy-SC)]Cl_2_ (**Ru3**, red) ([Ru] = 1/4 IC_50_). Data are represented as the mean fold increase over untreated cells used as a control (100%, dashed line).

Integrins are cell surface proteins responsible for cell adhesion to the extracellular matrix (ECM) and endothelial cells. The anchoring of cells through integrins to the ECM results in the transduction of signaling events that regulate many cellular processes such as cell survival, proliferation, and migration*.* Due to the observed changes in cell migration and adhesion properties, integrins might be the possible targets for Ru compounds. To elucidate whether the studied Ru complexes may be involved in regulating these monomeric or heterodimeric receptors, specific α and β integrin-binding assays were used to evaluate their effect on integrin translation and/or stability in MDA-MB-231 cells. In the performed study, the impact of Ru complexes was evaluated by monitoring changes in their accessibility to appropriate antibodies compared to untreated cells. Initial experiments using untreated MDA-MB-231 cells identified that these cells bound more efficiently to wells coated with the following array of integrin subunits/heterodimers monoclonal antibodies: α1, α2, α3, α5, β1, β4, αvβ3, αvβ5, and α5β1, and only those were further analyzed. Treatment with all three studied Ru compounds resulted in a decrease in functional α integrin subunits and αvβ3 heterodimer accessibility on the cell surface of the MDA-MB-231 cells (Figure 6A). In the case of integrins β4 subunit and α5β1 heterodimer, a significant down-regulation of cell surface production was observed only after treatment of MDA-MB-231 cells with **Ru2** (Figure 6B). On the contrary, treatment with **Ru3** and **Ru1** led to significantly elevated availability of the αvβ5 heterodimer and β4 subunit, respectively (Figure 6B).

Integrin expression in various types of human cancer was shown to be correlated with tumor cell invasion and migration potency, and among them, αvβ3, αvβ5, and α5β1 are well recognized.^24^ Inhibition of integrin functionality can affect contact with extracellular matrix components or endothelial cells (altering adhesion properties), disrupt signal transduction cascades that support migration, or both. Inhibition of α5β1 by **Ru2** can suppress MDA-MB-231 cell migration (Figure 5B) by affecting the migration machinery through signaling transduction. This type of inhibitory effect has already been reported.^25^ It was shown that in breast cancer, αVβ5 integrin is involved in cell migration through the regulation of urokinase that triggers cytoskeletal rearrangement and activation of protein kinase C.^26^ Only **Ru3** positively affected αVβ5 integrin accessibility, but the mechanism underlying the observed changes is unclear. αVβ3 integrin was demonstrated to be responsible for cell motility and trans-endothelial migration by supporting hypokinetic migration (relying on adhesion to the substrate) and activating matrix-degrading MMP-2.^22,27^ Therefore, the observed suppression of αVβ3 integrin functionally by all investigated Ru compounds explains their effect on the reduction of cell mobility, invasion, and transmigration (Figure 5) as well as re-adherence of cells (Figure 4). It should be noted that the superior effect of **Ru3** cannot be explained solely by its impact on integrins, suggesting the involvement of other targets.

### 2.5 Ru Complexes Induced Cell Adhesion Strengthening – Contributions from Focal Adhesion Assembly and Cytoskeleton Changes

**Figure 7:**
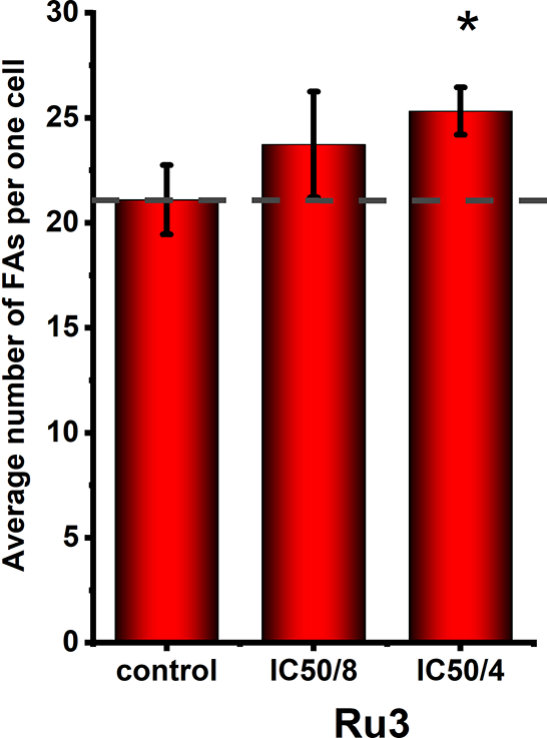
Quantification of focal adhesions (FAs) in MDA-MB-231 cells after 24 h of treatment with [Ru(dip)_2_(bpy-SC)]Cl_2_ (**Ru3**); untreated cells were used as control (dashed line). The bars represent the mean number of FAs per cell of ∼60 randomly selected adherent cells, calculated using ImageJ software. Focal adhesions were visualized by vinculin staining. *p < 0.05.

As discussed in Section 2.2, the studied compounds greatly impacted cell adhesion to cell culture plates (Figure 3). To gain better insight into the mechanism underlying the observed effect, the average number of focal adhesions (FAs) for MDA-MB-231 cells treated with **Ru3** for 24 h was counted (Figure S9). As shown in Figure 7, the number of FAs was significantly higher in **Ru3**-treated cells than the non-treated control.

**Figure 8:**
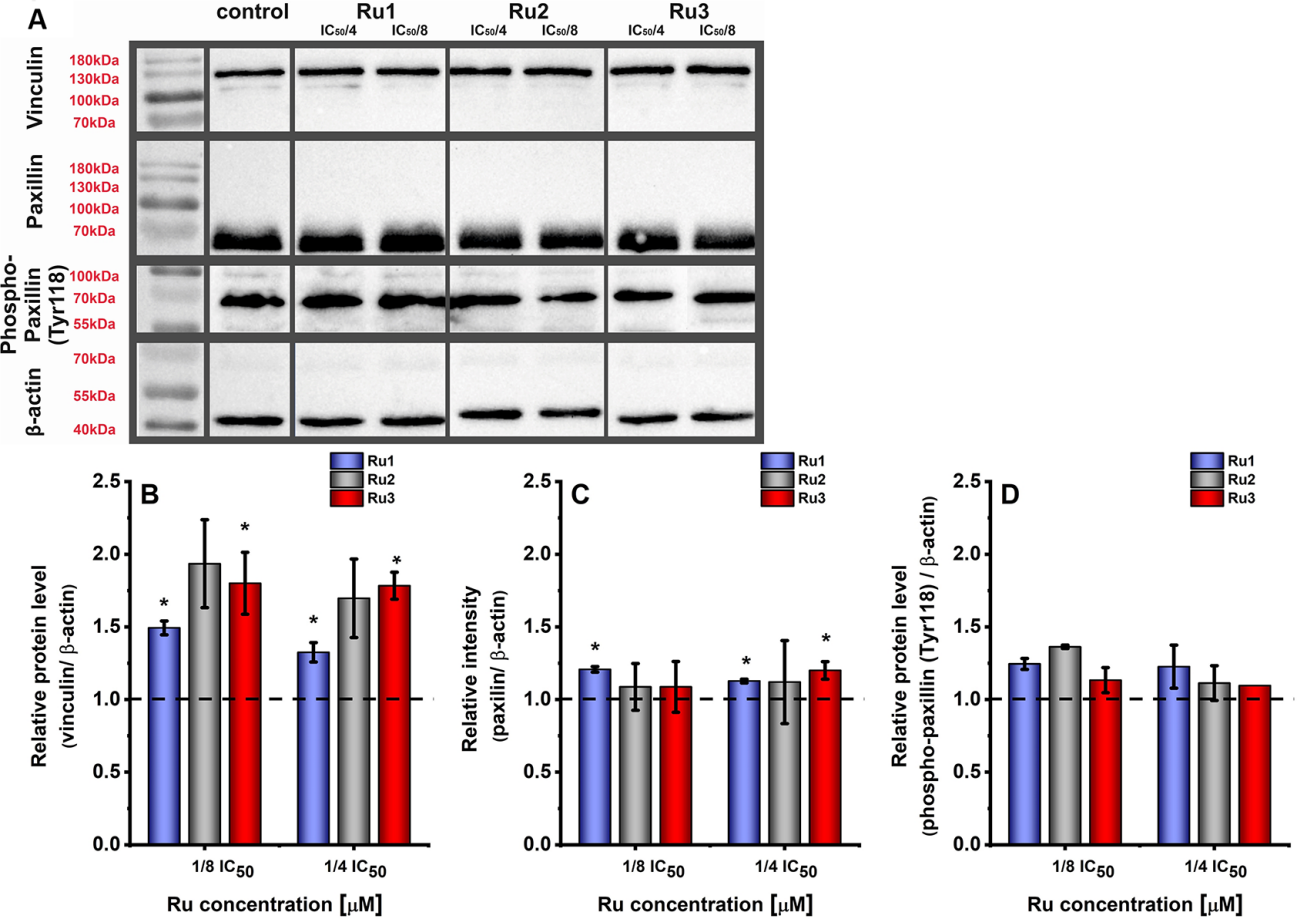
(A) Western blots of vinculin, paxillin, phospho-paxillin (Tyr118), and β-actin in MDA-MB-231 cells treated with the Ru complexes for 24 h (representative images). (B–D) A quantification of protein levels of focal adhesion components vinculin (B), paxillin (C), and phospho-paxillin (D) in MDA-MB-231 cells after 24 h of treatment with [Ru(dip)_2_(bpy)]Cl_2_ (**Ru1**, blue), [Ru(dip)_2_(bpy-NitroIm)]Cl_2_ (**Ru2**, gray), and [Ru(dip)_2_(bpy-SC)]Cl_2_ (**Ru3**, red). Untreated cells were used as control (dashed line). The expressions of proteins were calculated with respect to β-actin. Data are represented as mean ± SEM. *p < 0.05.

To gain further insight on the FA assembly, the expression of focal adhesion components vinculin and paxillin was evaluated for MDA-MB-231 cells treated with non-toxic doses of the investigated compounds. As shown in Figure 8A,B, the expression of vinculin was significantly increased by **Ru3**, while the lowest impact was observed for **Ru1**. More studies are needed to check whether Ru compounds also regulate vinculin activation. It was already shown that the recruitment of the focal adhesion structural protein vinculin could increase in adhesion strength after an initial binding.^27a^ Paxillin is another major component of focal adhesion, which may exert positive or negative effects on cell migration.^28^ Expression of paxillin increased significantly after treatment with **Ru1** and **Ru3** (Figure 8A,C). Since the function of paxillin is tightly regulated by phosphorylation,^29^ the amount of phosphorylated protein was additionally measured. As shown in Figure 8A,D, the 24 h treatment of MDA-MB-231 cells with the Ru complexes increased the efficiency of paxillin phosphorylation on the Tyr118 site.

Furthermore, the morphology of MDA-MB-231 cells treated with the Ru complexes was visualized by fluorescence staining of F-actin using a selective high-affinity probe ActinGreen 488 ReadyProbes (ThermoFisher Scientific). Representative images of cancer cells treated with the most active **Ru3** are presented in Figure S10. However, the observed changes were rather subtle (Figure S10) and did not allow one to form a definite statement regarding reorganization of F-actin filaments.

**Figure 9:**
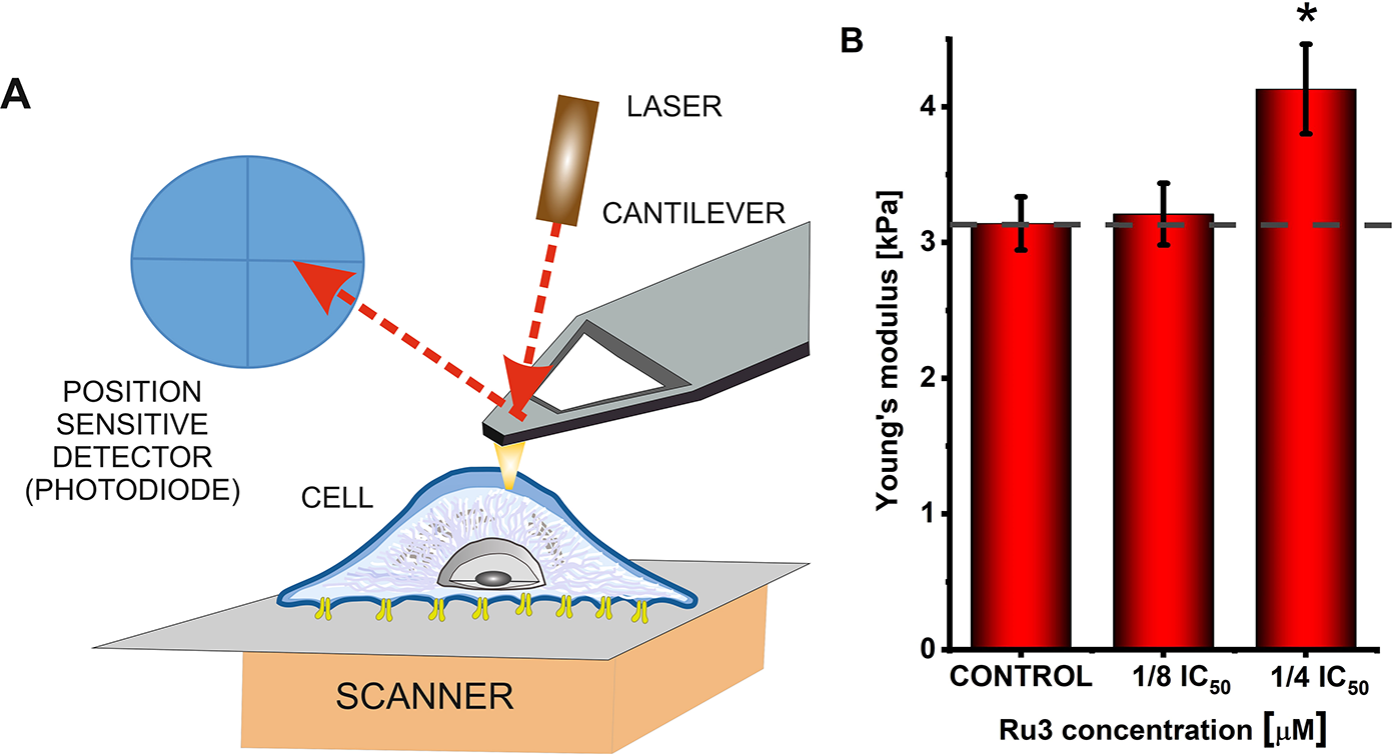
(A) Illustration of the main elements that constitute an atomic force microscope (AFM). (B) Elasticity of MDA-MB-231 cells measured after 24 h of treatment with [Ru(dip)_2_(bpy-SC)]Cl_2_ (**Ru3**). Young’s modulus is presented as a mean ± SEM for ∼80 randomly selected adherent cells with respect to the modulus of control cells (dashed line). *p < 0.05.

A cell elasticity study was conducted using atomic force microscopy (AFM) to obtain semiqualitative information regarding the reorganization of the cell cytoskeleton. A significant advantage of this study is that measurements were performed on living cells, a physiologically relevant environment. Details of this method were described earlier.^30^ Briefly, it is based on indenting the cell using a delicate cantilever with a probing tip mounted at the free end. Cantilever deflection is measured as a function of sample position (Figure 9A). It is converted into the relationship between the load force and indentation. The elastic properties of cells are quantified through Young’s (elastic) modulus. The AFM study of the mechanical properties of MDA-MB-231 cells after treatments with the most active compound, i.e., **Ru3**, revealed Young’s modulus increase of Ru-treated cells compared to the vehicle-treated control (Figure 9B). Higher moduli indicated larger rigidity of the cancer cells upon Ru treatments, which accounts for the decrease in cell deformability. It was suggested that the different F-actin organization might be responsible for these changes in cell elasticity.^31^ Numerous studies showed that cancer progression induced alterations in cell deformability in most cancers.^30^ Furthermore, some studies revealed a correlation between cell deformability and their metastatic potential.^32^

The observed impairment in cell migration and adhesion induced by the studied **Ru3** compound might arise from the alteration of the organization of the cancer cell cytoskeleton. The results are in line with our previous study, which revealed preferential accumulation of **Ru3** in the cytoskeleton fraction of cancer cells.^13^ The findings of the AFM elasticity study (Figure 9B) are also supported by the observed increase in vinculin expression after the treatment of cells with the Ru compounds (Figure 7D). Vinculin-deficient cancer cells are more deformable than the corresponding wild-type cells.^33^

## 3 Conclusions

Here, we report a family of Ru complexes that regulate the cell adhesion properties. On the one hand, they strengthen the attachment of cells in the plastic well; on the other hand, when cells are treated with the Ru complexes after their detachment, the re-adherence to plastic or endothelial monolayer is pronouncedly decreased. Adhesion formation and disassembly drive cell migration and play a crucial role in the propensity of cancer cells to invade. All studied compounds pronouncedly decreased migration, invasion, and transmigration – key metastasis processes. It is not without significance that the observed functional changes in cells are recorded for doses much lower than the cytotoxic dose. The most potent compound was **Ru3**, which exhibited significant changes in cell adhesion and motility. It demonstrated excellent uptake by cells and was particularly cytotoxic against highly invasive MDA-MB-231 cells. The insight on the molecular bases of the observed cell functional changes revealed that the studied compounds influence the integrin functionality and expression of the focal adhesion components vinculin and paxillin, resulting in an increased number of focal adhesion contacts. Furthermore, the observed increase in cell elasticity after treatment with **Ru3** may be related to its impact on the cytoskeleton. Taken together, it seems that studied Ru compounds can act simultaneously on several targets in the metastatic cascade that make them interesting candidates for their application as efficient antimetastatic agents. Applying hypoxic conditions, which are often encountered in solid tumors, did not change the cytotoxicity of the studied compounds in a significant manner, but their influence on cell adhesion and mobility was smaller.

We recognize that *in vitro* platforms used in the present study have many limitations in relation to *in vivo* experiments; however, they may provide clues as to the potential properties of the studied compounds that may deregulate or disrupt metastatic cascade.

## 4 Experimental Section

### 4.1 Materials

Unless otherwise stated, the reagents were purchased from Sigma-Aldrich. All complexes [Ru(dip)_2_(bpy)]Cl_2_ (**Ru1**), [Ru(dip)_2_(bpy-NitroIm)]Cl_2_ (**Ru2**), and [Ru(dip)_2_(bpy-SC)]Cl_2_ (**Ru3**) were prepared according to the published procedures. Their purity and identity were confirmed by HPLC (>95% pure) and ^1^H NMR together with HRMS analysis (HPLC traces and NMR spectrum are shown in the Supporting Information, Table S1).^27b,34^ Stock solutions of the Ru(II) polypyridyl complexes were prepared in DMSO.

### 4.2 Cell Culturing and Cytotoxicity Assay

The *in vitro* studies were conducted using two human breast cancer cell lines and two human melanoma cell lines as well as the human non-tumor immortalized keratinocyte cell line. Estrogen receptor-positive MCF-7 cells were cultured in EMEM medium supplemented with 2 mM glutamine, 1% Non-Essential Amino Acids (NEAA) (v/v), 10% fetal bovine serum (FBS) (v/v), and 1% penicillin–streptomycin solution (100 units/mL-100 μg/mL) (v/v) at 37 °C in a humidified atmosphere with 5% CO_2_ (v/v). Triple-negative MDA-MB-231 cells were cultured in L15 medium supplemented with 2 mM glutamine, 15% fetal bovine serum (FBS) (v/v), and 1% penicillin–streptomycin solution (100 units/mL-100 μg/mL) (v/v) at 37 °C in a humidified atmosphere. Low metastatic melanoma A375 cells were cultured in DMEM medium supplemented with 2 mM glutamine, 15% fetal bovine serum (FBS) (v/v), and 1% penicillin–streptomycin solution (100 units/mL-100 μg/mL) (v/v) at 37 °C in a humidified atmosphere with 5% CO_2_ (v/v). Highly metastatic melanoma A2058 cells were cultured in EMEM medium supplemented witch 2 mM glutamine, 1% Non-Essential Amino Acids (NEAA) (v/v), 10% fetal bovine serum (FBS) (v/v), and 1% penicillin–streptomycin solution (100 units/mL-100 μg/mL) (v/v) at 37 °C in a humidified atmosphere with 5% CO_2_ (v/v). Human non-cancerous immortalized keratinocytes cells HaCaT were cultured in DMEM medium supplemented with 10% fetal bovine serum (FBS) (v/v) and 1% penicillin–streptomycin solution (100 units/mL-100 μg/mL) (v/v) at 37 °C in a humidified atmosphere with 5% CO_2_ (v/v).

Hypoxic conditions were maintained in a humidified hypoxic chamber (Coy) filled with a gas mixture comprising 94% N_2_, 5% CO_2_, and, 1% O_2_. For the experiments performed in hypoxic conditions, cells were seeded under normal conditions and then moved to the hypoxic chamber for preincubation for at least 24 h. The medium intended to be used in hypoxic experiments was also preincubated in the hypoxic chamber for at least 24 h.

Cell viability upon treatment with the Ru(II) complexes was determined using the Alamar Blue assay. Cells were seeded into 96-well plates with a density of 3 × 10^4^ cells per cm^2^ in complete medium and cultured for 24 h. Subsequently, cells were incubated with various concentrations of the studied complexes for 24 h. Stock solutions of the Ru(II) complexes were prepared in DMSO. The final concentration of DMSO in cell culture was fixed at 0.1% (v/v). After 24 h of incubation, cells were washed with PBS and incubated in Alamar Blue solution for 3 h at 37 °C. Subsequently, the fluorescence was measured using a Tecan Infinite 200 microplate reader at 605 nm using 560 nm excitation light. The experiments were performed in triplicate and repeated three times. Results are presented as a mean value and standard error of the mean. IC_50_ parameters were determined using the Hill equation (OriginPro 2018).

### 4.3 Cellular Uptake of the Ru Compounds

Cellular uptake of the Ru complexes was determined using the all-tested cell line. Cells were seeded in 6-well plates with a density of 4 × 10^4^ cells per cm^2^ in a complete medium and cultured for 1 day. Next, cells were incubated with non-toxic concentrations of the Ru complex (either 1/8 or 1/4 of IC_50_) for 24 h. Subsequently, the incubated cells were washed, detached by trypsin treatment, counted, and centrifuged. The supernatant was removed, and cells were digested in concentrated nitric acid overnight at room temperature. The solutions were then diluted with Millipore water to a final nitric acid concentration of 1%. Samples were analyzed using inductively coupled plasma mass spectrometry (ICP-MS, NexION 2000C, PerkinElmer). The results were calculated as the Ru concentration per cell. The experiments were repeated three times.

### 4.4 Trypsin Resistance Assay

The influence of the susceptibility of cells to detachment upon incubation with the Ru(II) complexes was evaluated by checking their resistance to trypsin treatment. Cells were seeded into 96-well plates with a density of 3 × 10^4^ cells per cm^2^ in a complete medium and cultured for 24 h. Then, cells were incubated with various concentrations of Ru(II) complexes (either 1/8 or 1/4 of IC_50_) for 24 h. Subsequently, the cells were washed, and 30 μL of trypsin solution (0.05% for A2058 and MCF-7, 0.01% for A375 and MDA-MB-231) was added to each well for 5 min of incubation at 37 °C. The cells were then washed with PBS, and an Alamar Blue assay was performed to quantify the adherent cells. The received results were normalized with appropriate wells without trypsin treatment to exclude the possible toxicity of the studied compounds and presented as a percentage of control cells. For melanoma cell lines, experiments were performed under normoxic and hypoxic conditions. The experiments were carried out in triplicate, and each experiment was repeated five times to obtain the mean values and the standard error of the mean.

### 4.5 Re-adherence to the Substrate

The effect of the studied complexes on the adhesion properties of cancer cells was also examined by evaluating the ability of the treated cells to re-adhere. Cells were seeded into 6-well plates with a density of 3 × 10^4^ cells per cm^2^ in a complete medium and cultured for 24 h. Then, the medium was removed, and various concentrations of the studied Ru(II) complexes were added and incubated with the cells for 24 h. Subsequently, cells were washed and incubated with a fresh portion of PBS without Mg and Ca ions for 20 min. Then, the cells were detached with a cell dissociation solution, counted, and seeded into 96-well plates with a density of 6 × 10^4^ cells per cm^2^. The plates were incubated for 1 h in a humidified atmosphere at 37 °C and then were washed with PBS to remove non-adherent cells. A resazurin assay was performed to quantify the adherent cells. Detachment of MCF-7 cells treated with **Ru3** was not possible by using the cell dissociation solution, so instead a trypsin solution (0.05%) was used. For melanoma cell lines, experiments were performed under normoxic and hypoxic conditions. The experiments were carried out in triplicate, and each experiment was repeated five times to calculate the mean values and the standard error of the mean.

To evaluate re-adhesion of cancer cells to the monolayer of endothelial cells, HMEC-1 cells were seeded in a 24-well plate with a density of 4 × 10^4^ cells per cm^2^ in a complete medium 2 days before the experiments. Cancer cells were seeded in 6-well plates with a density of 3 × 10^4^ cells per cm^2^ in a complete medium and cultured for 24 h. The medium was removed, and various concentrations of the studied Ru(II) complexes were added and incubated with the cells for another 24 h. After incubation, cancer cells were washed and detached with trypsin solution (0.05%). The cancer cells were labeled with CellTracker Green CMFDA Dye (Invitrogen, ThermoFisher Scientific) according to the manufacturer’s instructions and counted. Labeled cancer cells were added to an endothelial cell monolayer at a ratio of cancer cells to endothelial cells of 1:1. Cells were incubated in a CO_2_ incubator for 1 h in serum free medium and then gently washed with PBS twice (to detach non adherent cells). The cells were detached with trypsin and analyzed by flow cytometry. The experiment was carried out in triplicate and repeated five times.

### 4.6 Migration, Invasion, and Transmigration Assays

The cell migration assay was tested using a commercial Transwell insert (8 μm pore size, Corning). Before the experiment, A2058 and MCF-7 cells were starved with a serum-free medium for 24 h. A375 and MDA-MB-231 cells were tested without prior incubation in a medium without FBS. Next, 5 × 10^4^ (for A375 and MDA-MB-231) or 1 × 10^5^ (for A2058 and MCF-7) cells in a serum-free medium were added to the upper chamber and a medium with 20% FBS was added to the lower chamber. The studied ruthenium complexes at various concentrations were added to both inserts and wells. After 16 h of incubation, the inserts were washed with PBS and the cells on the membrane were first fixed with 10% formalin and then stained with 0.5% crystal violet. After that, non-migrated cells were removed from the upper surface of the membrane using a cotton swab. Crystal violet was then dissolved in methanol, and absorbance was measured using a Tecan Infinite 200 microplate reader at 590 nm with 700 nm as a reference wavelength. The experiments were performed in duplicate, and each experiment was repeated twice to obtain the mean values and the standard error of the mean. The results are presented as a percentage of control cells not treated with the Ru compounds.

In the invasion assay, the Transwell Boyden chamber was pre-coated with Matrigel (1 mg/mL for MDA-MB-231 or 2 mg/mL for A375 cells) for 2 h at 37 °C. The rest of the procedure was the same as for the migration assessment.

In the transmigration assay, first, 3 × 10^4^ HMEC-1 cells in full-serum were seeded in the upper chamber and allowed to form a monolayer over 24 h. Next, endothelial cells were activated with 10 μg/mL TNFα for 15 min. Parallel tumor cells were seeded into 6-well plates with a density of 3 × 10^4^ cells per cm^2^ in complete medium and cultured for 24 h. Then, the medium was removed. Various concentrations of the studied Ru(II) complexes were added and incubated with the cells for 24 h. Afterward, the cells were washed, detached with trypsin, counted, and stained with CellTracker Green CMFDA Dye. A tumor cell suspension was added above the endothelial monolayer with a density of 1 × 10^5^ in a serum-free medium. Medium with 20% FBS was introduced into the lower chamber and incubated for 16 h. After this time, the inserts were washed with PBS, and non-invasive cells were removed from the upper surface of the membrane using a cotton swab, and the fluorescence was measured using a Tecan Infinite 200 microplate reader at 535 nm using 485 nm excitation light. The experiments were performed in duplicate, and each experiment was repeated twice to obtain the mean values and the standard error of the mean. Results are presented as a percentage of control cells.

For melanoma cell lines, migration, invasion, and transmigration experiments were performed under normoxic and hypoxic conditions.

### 4.7 Integrin Binding Assay

The influence of the complexes on the expression of specific integrins on the cell surface was assessed using an Alpha/Beta Integrin-Mediated Cell Adhesion Array Combo Kit (ECM535 Millipore). MDA-MB-231 cells were grown to confluence in 75 cm^2^ flasks. Then, the cells were washed and detached with a cell dissociation solution. In the next step, cells were counted and the suspension was prepared with a density of 1 × 10^6^ cells per 1 mL in L15 medium. The suspension was divided into portions, and Ru(II) compounds in DMSO were added to obtain an appropriate concentration (the DMSO concentration was kept at 0.1% v/v). 100 μL of such prepared cell suspensions was added to the integrin antibody-coated and control wells and incubated for 2 h at 37 °C. The unbound cells were then washed off, and the adherent cells were stained with CyQuant GR dye. Subsequently, the fluorescence was measured using a Tecan Infinite 200 microplate reader at 530 nm using 485 nm excitation light. The data were combined from three independent experiments. Each sample was assayed twice. Data are represented as the mean percentage of control and the standard error of the mean.

### 4.8 Cell Lysis and Immunoblotting

Expression levels of focal adhesion components vinculin, paxillin, and phospho-paxillin (Tyr118) were measured by the Western blot technique in MDA-MB-231 protein extracts. After 24 h of incubation with the studied Ru(II) complexes, cells were rinsed twice with PBS (4 °C) and lysed on ice with RIPA lysis buffer. The lysates were purified by centrifugation, and protein concentrations were determined by the Bradford method using bovine serum albumin as the standard. Then, to 30 μL of each lysate sample was added 10 μL of sample buffer (0.5 M TRIS, 10% glycerol, β-mercaptoethanol, 10% SDS). The samples were heated at 95 °C for 5 min. The obtained protein extracts were subjected to pre-electrophoresis (60 V/25 min) and electrophoresis (170 V/50 min) on a 12.5% SDS polyacrylamide gel at room temperature using a PowerPac Basic Power Supply (Bio-Rad, Inc., Hercules, CA, USA). PageRuler Prestained Protein Ladder (Thermo Fisher Scientific) was used to determine the approximate molecular weights of resolved proteins. A wet electrotransfer was carried out for 2 h at a constant current of 200 mA to transfer the separated proteins to a polyvinylidene difluoride (PVDF) membrane (Bio-Rad, Inc., Hercules, CA, USA). Furthermore, the membrane was probed with a primary antibody overnight at 4 °C, namely, mouse monoclonal anti-paxillin antibody (dilution 1:250; Thermo Fisher Scientific, catalogue number AH00492), rabbit monoclonal anti-vinculin antibody (dilution 1:500; Thermo Fisher Scientific, catalogue number 42H89L44), rabbit polyclonal anti-phospho-paxillin (Tyr118) antibody (dilution 1:1000; Thermo Fisher Scientific, catalogue number 44-722G), or mouse monoclonal anti-β-actin antibody (dilution 1:1000; Thermo Fisher Scientific, catalogue number AM4302), and a secondary antibody for 2 h at room temperature, namely, goat anti-mouse antibody (dilution 1:10,000; Thermo Fisher Scientific, catalogue number G21040) for paxillin and β-actin detection or goat anti-rabbit antibody (dilution 1:10,000; Thermo Fisher Scientific, catalogue number G21234) for vinculin and phospho-paxillin detection. Reactive protein was detected using GE Healthcare Amersham ECL Prime Western Blotting Detection Reagent (GE Healthcare Inc., Chicago, IL, USA). Data were collected by a ChemiDoc XRS+ Imaging System (Bio-Rad, Inc., Hercules, CA, USA) and analyzed with Image Lab v. Software 6.1.0 software (Bio-Rad, Inc., Hercules, CA, USA). The analysis was done using three independent biological repetitions. β-Actin was used for normalization.

### 4.9 Fluorescence Imaging

The cultured cells were gradually fixed with PFA (10 min 1% and 10 min 2%) and permeabilized for 5 min in PBS containing 0.2% Triton-X100. For focal adhesion (FA) staining, anti-vinculin FITC conjugated mouse monoclonal antibody (dilution 1:50; F7053 Sigma Aldrich) was used and incubated for 1 h at room temperature followed by imaging with an Olympus IX83 microscope (λ_ext_ at 470 nm, λ_em_ at 525). Data represents the mean number of FAs per one cell of ∼60 randomly selected adherent cells, calculated using the ImageJ software. According to the attached protocol, cytoskeletons were stained using ActinGreen 488 Ready Probes (Life Technologies R37110). Images were taken using an Olympus IX83 microscope (λ_ext_ at 470 nm, λ_em_ at 525).

### 4.10 Atomic Force Microscopy - Elasticity Measurements

To assess the relative changes in mechanical properties of MDA-MB-231 cells after **Ru3** treatments, AFM experiments were carried out using an XE-120 (Park System, South Korea) with a combined Olympus IX71 inverted optical microscope (Olympus, Japan). The optical microscope was used to move and align cantilevers above the cells. Measurements were performed using commercially available silicon nitride cantilevers with a nominal spring constant of 0.03 N/m, open-angle of 36°, and tip radius of 10 nm (PNP-TR-B, Nanoworld). Prior to the experiments, the spring constant of the cantilever was measured using the thermal noise calibration. MDA-MB-231 cells were seeded into 6-well plates on glass coverslips (15 mm × 15 mm) with a density of 4 × 10^3^ cells per cm^2^ in complete medium and cultured for 24 h. This small seeding density was chosen to avoid the influence of neighboring cells on the measurements made. Then, cells were incubated with various concentrations of **Ru3** (either 1/8 or 1/4 of IC_50_) for 24 h. After incubation, cells were washed with DPBS, and the coverslip with cells was transferred to a “liquid cell” placed on an AFM scanner. The measurements were conducted in the basal cell culture medium at room temperature. An average experiment lasted no more than 2 h to preserve the viability of the cells. Cells were indented approximately over the nuclear region of individual cells. The 25 force curves were recorded over a scan area of 25 μm^2^. During each experiment, 20 cells were measured for each studied condition. The experiments were repeated four times. The force curves were converted into force versus indentation curves and further analyzed. For Young’s modulus determination, the probe shape was assumed to be conical. The average values of Young’s modulus were calculated and presented as the mean value with the standard error of the mean.

### 4.11 Statistical Analysis

For *in vitro* experiments, all data were expressed as the mean and standard error of the mean (SEM). For statistical analysis between the control group and the experiment groups, one-way analysis of variance (ANOVA) was performed, and the Mann–Whitney U test was performed for statistical analysis when the data did not accord with the homogeneity of variance (Statistica 13.3). Probabilities of *p* < 0.05 were considered statistically significant. The following notification is used: * *p* < 0.05.

## Supporting Information Available

Purity confirmation (Table S1), trypsin resistance assay (Figure S1), re-adhesion assay (Figure S2), migration, invasion, and transmigration assays (Figures S3–S8), focal adhesion detection (Figure S9), and F-actin filament visualization (Figure 10) (PDF)
Molecular formula strings (CSV)

## Abbreviations

AFM: atomic force microscopy
bpy: 2,2′-bipyridine
bpy-NitroIm: 4-[3-(2-nitro-1H-imidazol-1-yl)propyl]
bpy-SC: 5-(4-{4′-methyl-[2,2′-bipyridine]-4-yl}but-1-yn-1-yl)pyridine-2-carbaldehyde semicarbazone
CAM: cell adhesion molecules
dip: 4,7-diphenyl-1,10-phenanthroline
DMEM: Dulbecco’s modified Eagle medium
ECM: extracellular matrix
EMEM: Eagle’s minimum essential medium
ER: estrogen receptor
Fac: focal adhesion contacts
FBS: fetal bovine serum
HER2: human epidermal growth factor receptor
ICP-MS: inductively coupled plasma mass spectrometry
LDM: low-dose metronomic chemotherapy
MTD: maximum tolerated doses
NEAA: non-essential amino acids
PFA: paraformaldehyde
PR: progesterone receptor
PVDF: polyvinylidene difluoride
SEM: standard error of the mean
