# Supplementary material for: Inhibition of Metastasis by Polypyridyl Ru(II) Complexes through Modification of Cancer Cell Adhesion – In Vitro Functional and Molecular Studies
Source: J Med Chem. 2022 Jul 27;65(15):10459–70. doi: 10.1021/acs.jmedchem.2c00580 (PMC9376949; doi:10.1021/acs.jmedchem.2c00580)
Supplement: Supplementary file 1 — jm2c00580_si_001.pdf [file jm2c00580_si_001.pdf]

Supporting Information

Inhibition of metastasis by polypyridyl Ru(II) complexes through modification of cancer cell adhesion – *in vitro* functional and molecular studies

Ilona Gurgul[#,1], Ewelina Janczy-Cempa[#,1], Olga Mazuryk*[,1], Małgorzata Lekka[2], Michał Łomzik[1,3], Franck Suzenet[4], Philippe C. Gros[5], Małgorzata Brindell*[,1]

[1]*Faculty of Chemistry, Jagiellonian University in Krakow, Gronostajowa 2, 30-387 Krakow, Poland;*
[2]*Department of Biophysical Microstructures, Institute of Nuclear Physics, Polish Academy of Sciences, PL-31342 Krakow, Poland*
[3]*Department of Organic Chemistry, Faculty of Chemistry, University of Łódź, ul. Tamka 12, 91-403 Łódź, Poland*
[4]*Institute of Organic and Analytical Chemistry, University of Orléans, UMR-CNRS 7311, rue de Chartres, BP 6759, 45067 Orléans Cedex 2, France*
[5]*Université de Lorraine, CNRS, L2CM, F-54000 Nancy, France*

Corresponding authors: olga.mazuryk@uj.edu.pl, malgorzata.brindell@uj.edu.pl
[#]both authors contributed equally to this work

Content

1. **Purity confirmation** 2
2. **Cell adhesion properties – trypsin resistance assay** 3
3. **Re-adhesion assay** 4
4. **Migration, Invasion, and transmigration** 5
   a. **Migration** 5
   b. **Invasion** 9
   c. **Transmigration** 9
5. **Focal adhesions (FAs) detection** 10
6. **F-actin filaments visualization** 10

1. Purity confirmation

Compound	HPLC or NMR+HRMS analysis
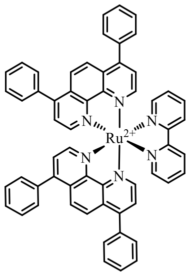	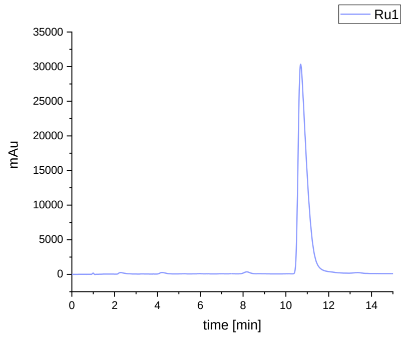
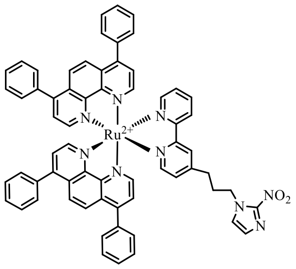	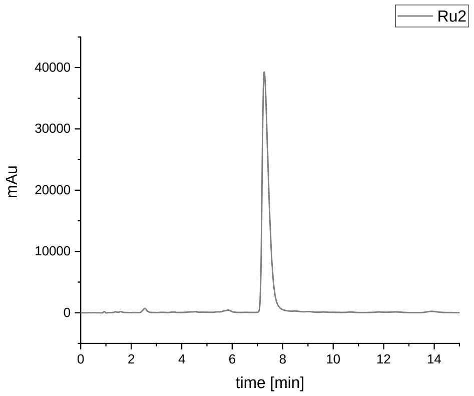
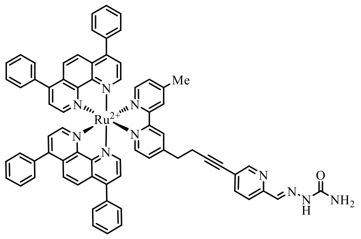	HRMS: m/z: Calculated for $C_{70}H_{52}N_{10}ORu$: m/z 575.1679 (M^+), Found 575.1658 (M^+).<br>^{1}H NMR (600 MHz, CD_3CN) □ 2.55 (s, 3H), 2.95 (t, *J* =6 Hz, 2H), 3.13 (t, *J*=6 Hz, 2H), 7.04 (s, 1H), 7.22 (d, *J*=6 Hz, 1H), 7.37 (dd, *J*=15.6 Hz, 6 Hz, 2H), 7.57-7.63 (m, 20H), 7.67 (d, *J*=5.4 Hz, 2H), 7.73 (dd, *J*=5.4 and 2.4 Hz, 2H), 7.79 (t, *J*=5.4 Hz, 1H), 8.13-8.20 (m, 6H), 8.23 (d, *J*=5.4 Hz, 1H), 8.26 (d, *J*=5.4 Hz, 1H), 8.33 (d, J=5.4 and 3.6 Hz, 1H), 8.80 (d, J=12.6 Hz, 1H), 8.90 (d, J=12.6 Hz, 1H) ppm<br>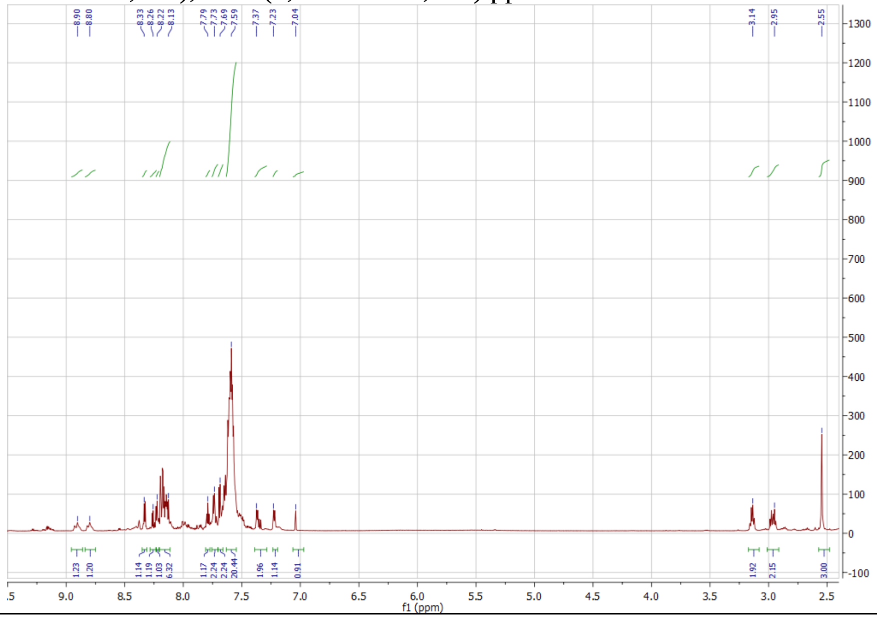

2. Cell adhesion properties – trypsin resistance assay

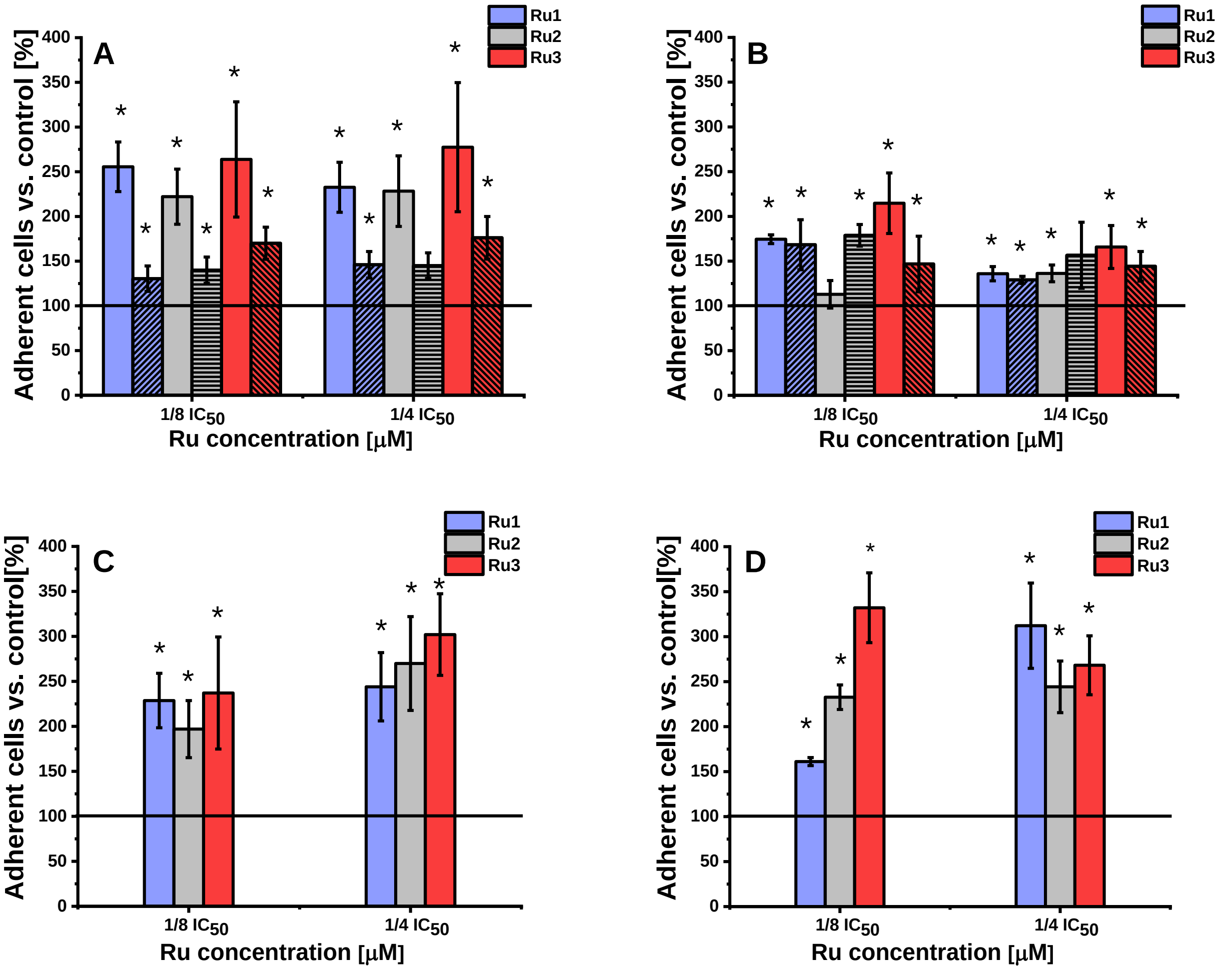

Fig S1. A) A375, **B) A2058, C) MCF-7** and **D) MDA-MB-231** cell adhesion evaluated as the percentage of remained adherent cells upon controlled trypsin treatment. Cells were incubated with **$[Ru(dip)_2(bpy)]Cl_2$ (Ru1,** blue**), $[Ru(dip)_2(bpy\text{-}NitroIm)]Cl_2$ (Ru2,** gray**) and $[Ru(dip)_2(bpy\text{-}SC)]Cl_2$ (Ru3,** red**)** under normoxic (filled bar) and hypoxic (dashed bar) conditions. Untreated cells were used as control (100%). *$p<0.05$

3. Re-adhesion assay

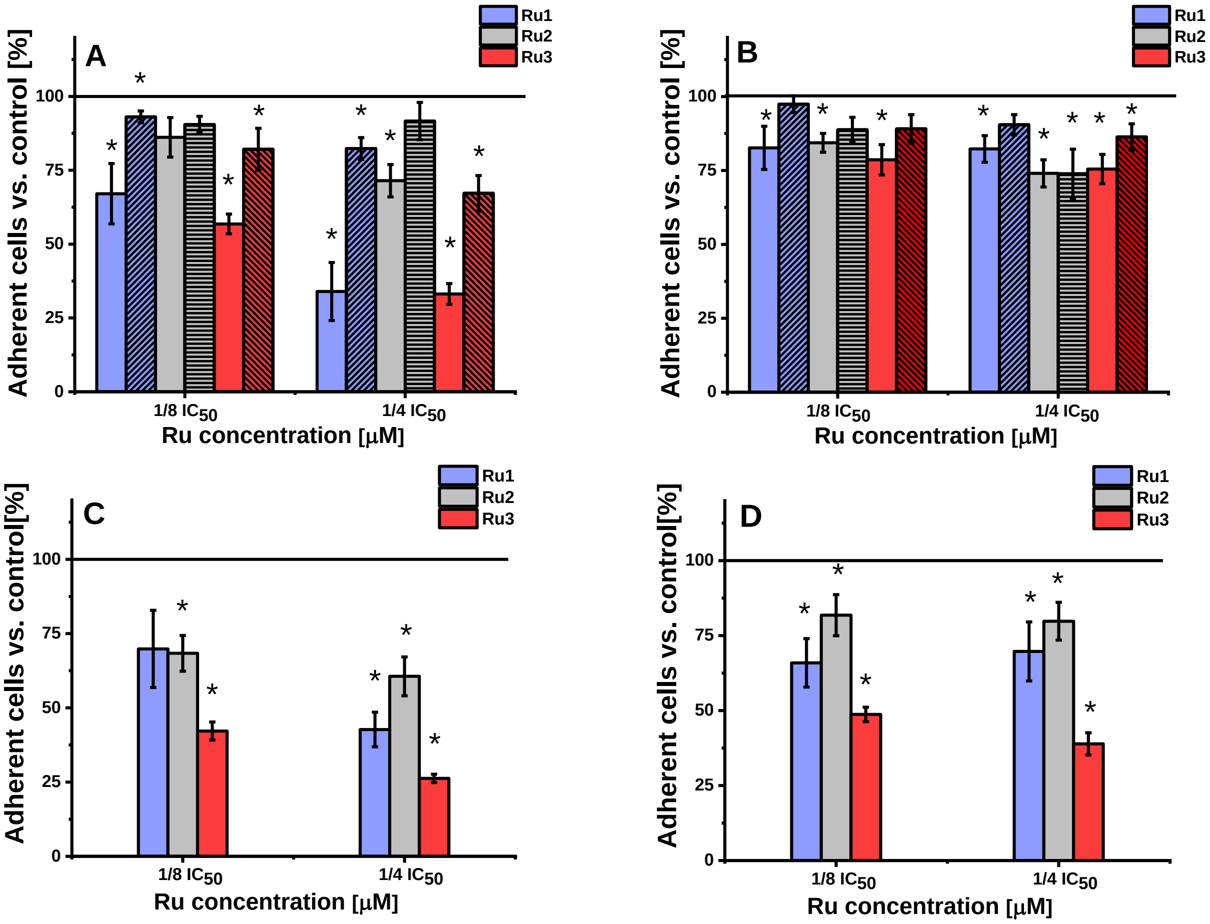

Fig S2. A) A375, B) A2058, C) MCF-7 and **D) MDA-MB-231** cells' ability to re-adhere, measured after 24 h incubation with **$[Ru(dip)_2(bpy)]Cl_2$ (Ru1,** blue**), $[Ru(dip)_2(bpy\text{-}NitroIm)]Cl_2$ (Ru2,** gray**) and $[Ru(dip)_2(bpy\text{-}SC)]Cl_2$ (Ru3,** red**)**. under normoxic (filled bar) and hypoxic (dashed bar) conditions. Untreated cells were used as control (100%). *$p<0.05$

4. Migration, Invasion, and transmigration

a. Migration

Method 1: Direct incubation of cells with ruthenium complexes on the inserts

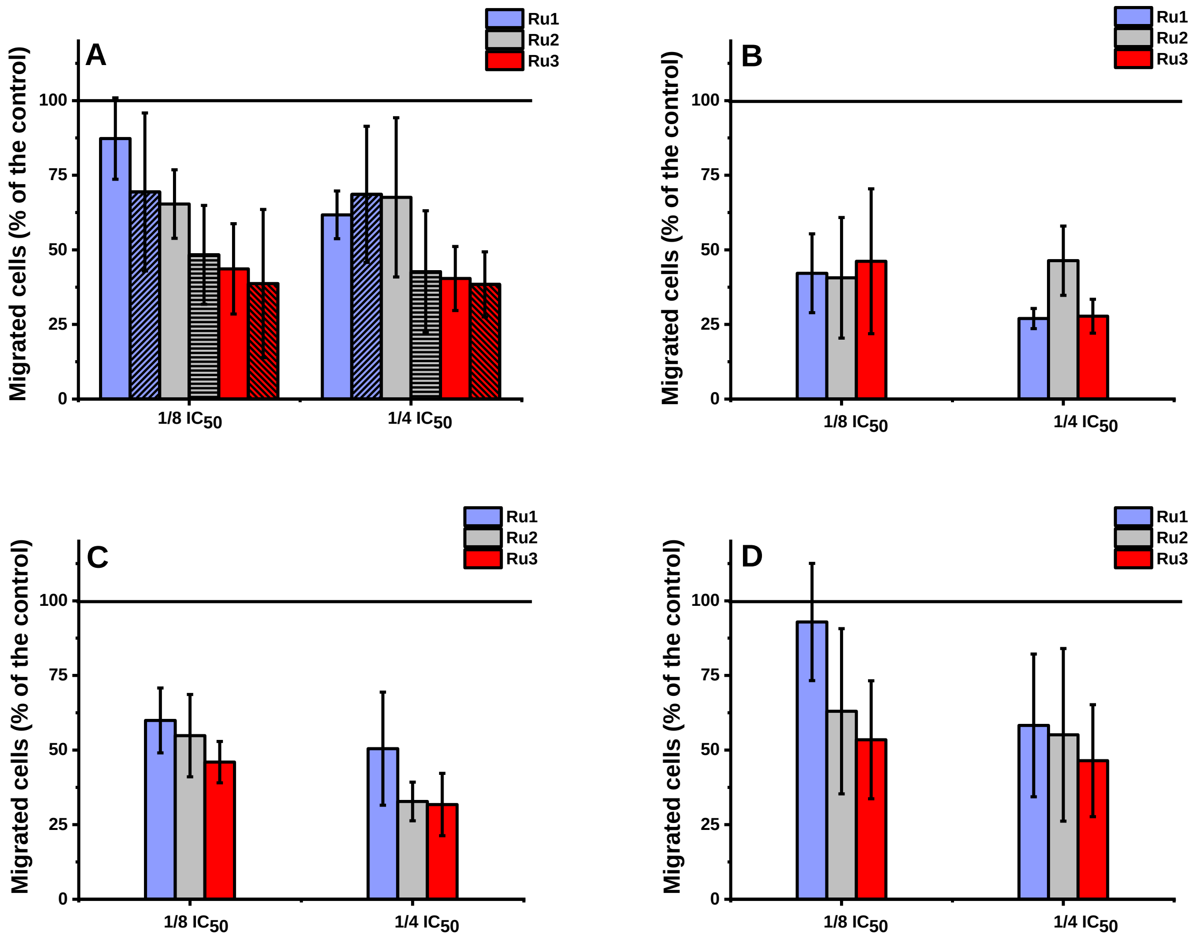

Fig S3. Effects of **[Ru(dip)$_2$(bpy)]Cl$_2$ (Ru1,** blue**), [Ru(dip)$_2$(bpy-NitroIm)]Cl$_2$ (Ru2,** gray) **and [Ru(dip)$_2$(bpy-SC)]Cl$_2$ (Ru3,** red**)** on the migration of ***A) A375, B) A2058, C) MCF-7 and D) MDA-MB-231*** under normoxic (filled bar) and hypoxic (dashed bar) conditions. Untreated cells were used as control (100%).

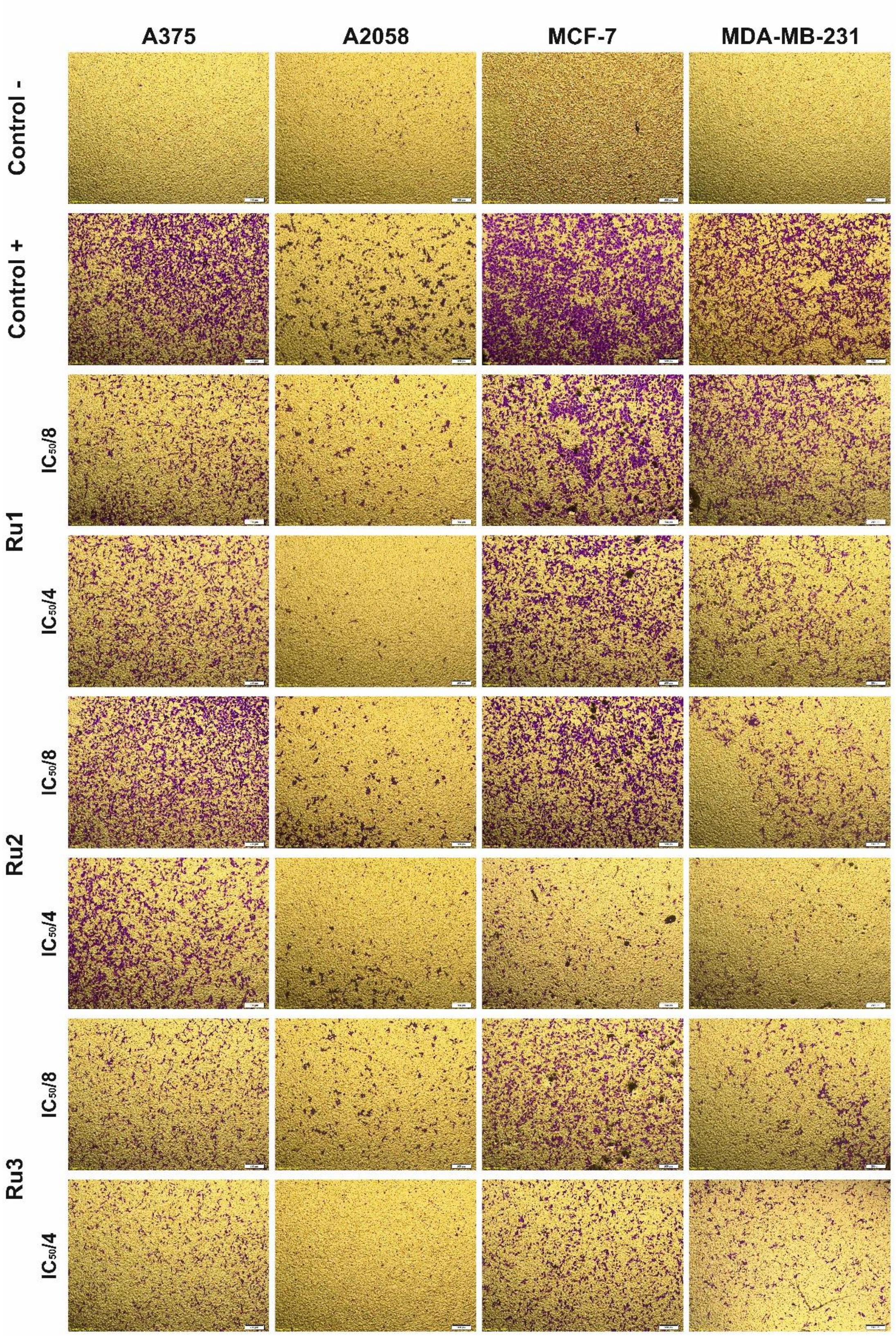

Fig S4. Representative images of the effect of direct incubation of Ru complexes (**[Ru(dip)$_2$(bpy)]Cl$_2$** (**Ru1**), **[Ru(dip)$_2$(bpy-NitroIm)]Cl$_2$** (**Ru2**) and **[Ru(dip)$_2$(bpy-SC)]Cl$_2$** (**Ru3**)) on the migration of cancer cells (A375, A2058, MCF-7 and MDA-MB-231 cell lines) under normoxic conditions. Control-/+ denotes untreated cells without/with a chemoattractant.

Method 2: Pre-incubation of cells with ruthenium complexes

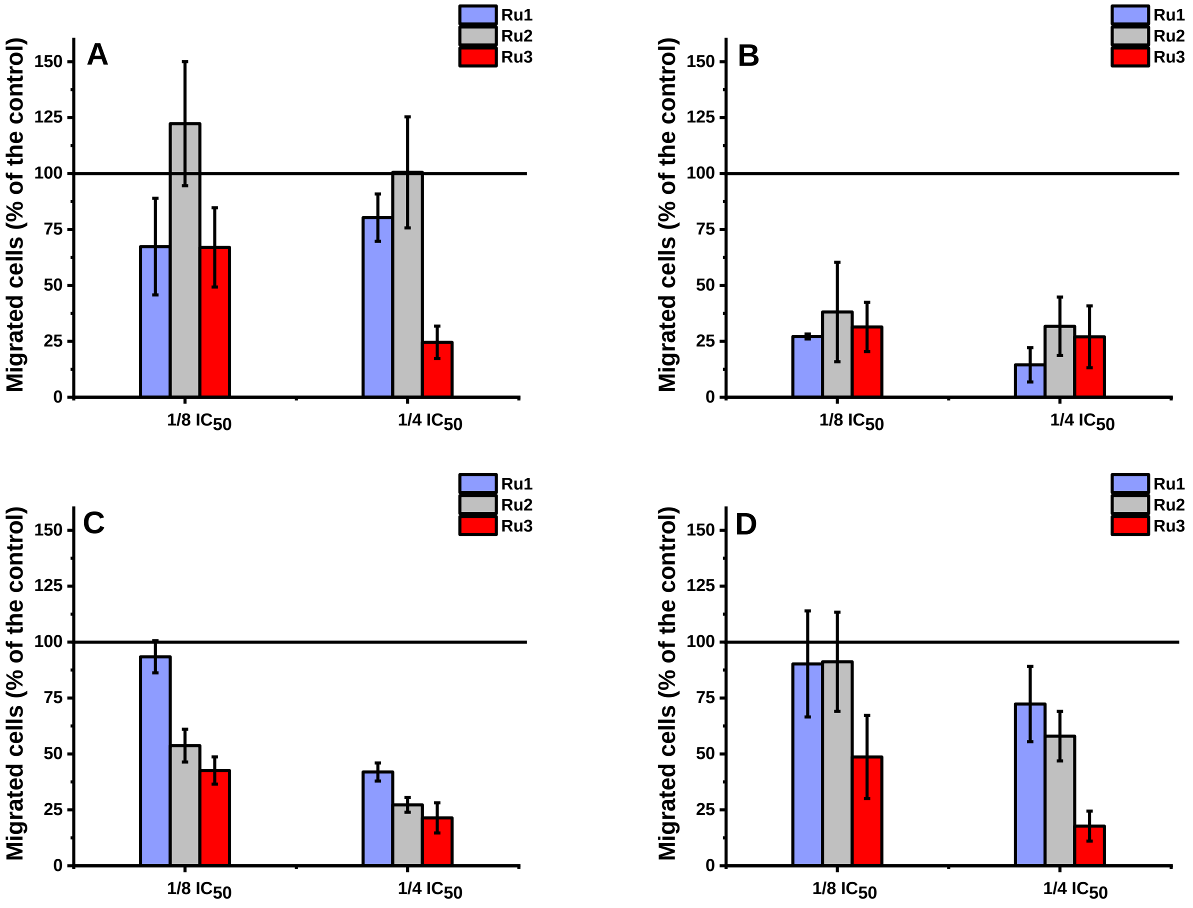

Fig S5. Effects of **$[Ru(dip)_2(bpy)]Cl_2$ (Ru1,** blue**), $[Ru(dip)_2(bpy\text{-}NitroIm)]Cl_2$ (Ru2,** gray**) and $[Ru(dip)_2(bpy\text{-}SC)]Cl_2$ (Ru3,** red**)** on the migration of ***A) A375, B) A2058, C) MCF-7*** *and* ***D) MDA-MB-231*** under normoxic (filled bar) and hypoxic (dashed bar) conditions. Untreated cells were used as control (100%).

Cells were seeded into a 6-well plate with the density of 3×10^4 cells per cm^2 in complete medium and cultured for 24 h. Then, the medium was removed and various concentrations of the studied Ru(II) complexes were added and incubated with the cells for 24 h. Afterwards, the cells were washed, detached with trypsin, counted and seeded into an insert with the density of 5×10^4 (for A375 and MDA-MB-231) or 1×10^5 (for A2058 and MCF-7) cells in a serum-free medium. The medium with 20% FBS was introduced into the lower chamber and incubated for 16 h. After this time, inserts were washed with PBS, and cells on the membrane were first fixed with 10% formalin and then stained with 0.5 % crystal violet. After that, non-migrated cells were removed from the upper surface of the membrane using a cotton swab. Next, the crystal violet was dissolved in methanol, and the absorbance was measured using a Tecan Infinite 200 microplate reader at 590 nm with 700 nm as a reference wavelength. Experiments were performed in duplicate, and each experiment was repeated two times to obtain mean values and standard error of the mean. Results are presented as a percentage of control cells, which were not treated with Ru compounds.

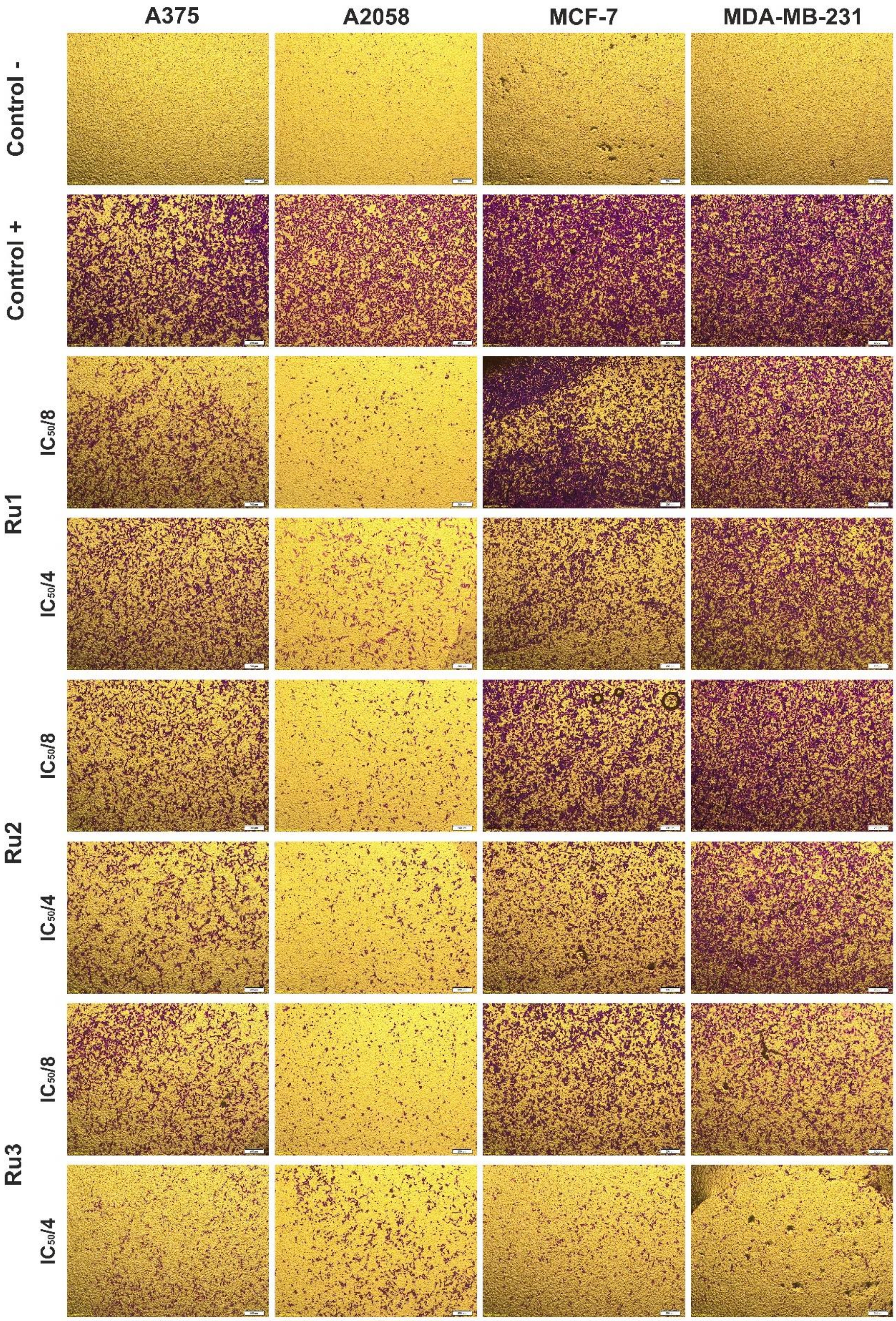

Fig S6. Representative images of the influence on the migration of cancer cells (A375, A2058, MCF-7 and MDA-MB-231 cell lines) pre-incubated with Ru complexes (**[Ru(dip)$_2$(bpy)]Cl$_2$** (**Ru1**), **[Ru(dip)$_2$(bpy-NitroIm)]Cl$_2$** (**Ru2**) and **[Ru(dip)$_2$(bpy-SC)]Cl$_2$** (**Ru3**)) under normoxic conditions. Control-/+ denotes untreated cells without/with a chemoattractant.

b. Invasion

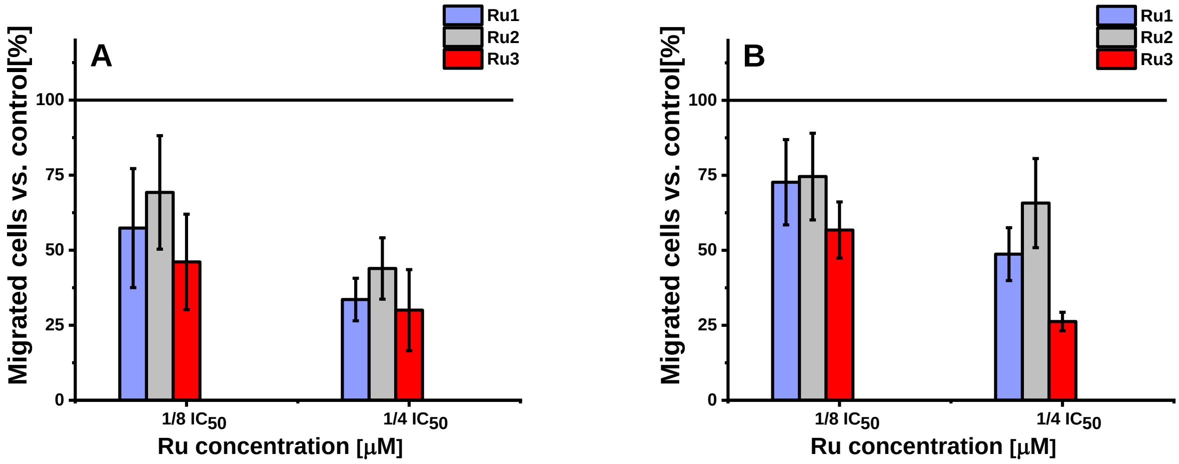

Fig. S7. Effects of **[Ru(dip)$_2$(bpy)]Cl$_2$ (Ru1,** blue**), [Ru(dip)$_2$(bpy-NitroIm)]Cl$_2$ (Ru2,** gray**) and [Ru(dip)$_2$(bpy-SC)]Cl$_2$ (Ru3,** red**)** on the invasion of ***A) A375 and B) MDA-MB-231.***

c. Transmigration

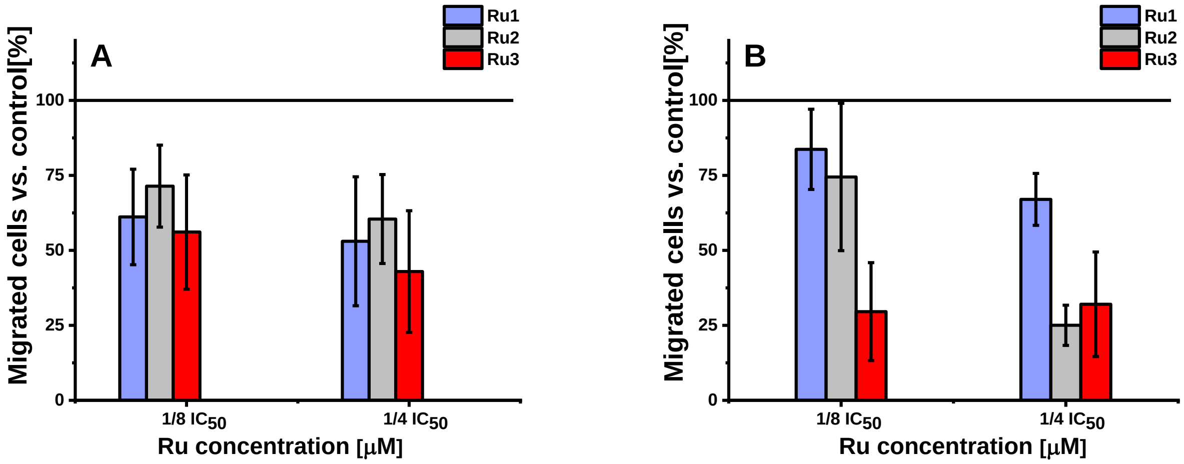

Fig. S8. Effects of **[Ru(dip)$_2$(bpy)]Cl$_2$ (Ru1,** blue**), [Ru(dip)$_2$(bpy-NitroIm)]Cl$_2$ (Ru2,** gray**) and [Ru(dip)$_2$(bpy-SC)]Cl$_2$ (Ru3,** red**)** on the transmigration of ***A) A375 and B) MDA-MB-231.***

5. Focal adhesions (FAs) detection

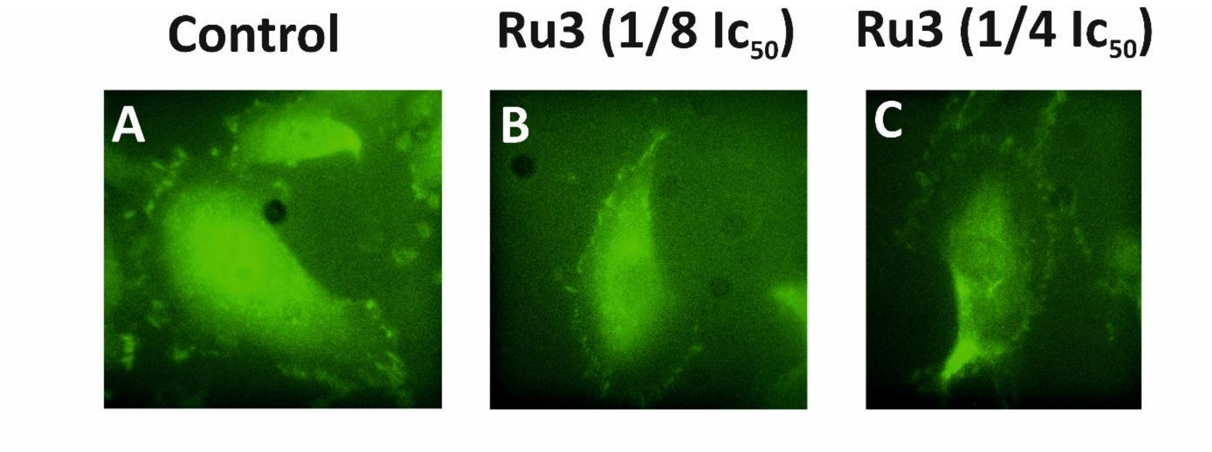

Fig. S9. Representative fluorescent images for focal adhesions (FAs) detection. MDA-MB-231 cells treated with different concentrations of **[Ru(dip)$_2$(bpy-SC)]Cl$_2$ (Ru3**) for 24 h (**B**, **C**). Cells treated with vehicle were used as control (**A**). Focal adhesion points were visualized by vinculin staining.

6. F-actin filaments visualization

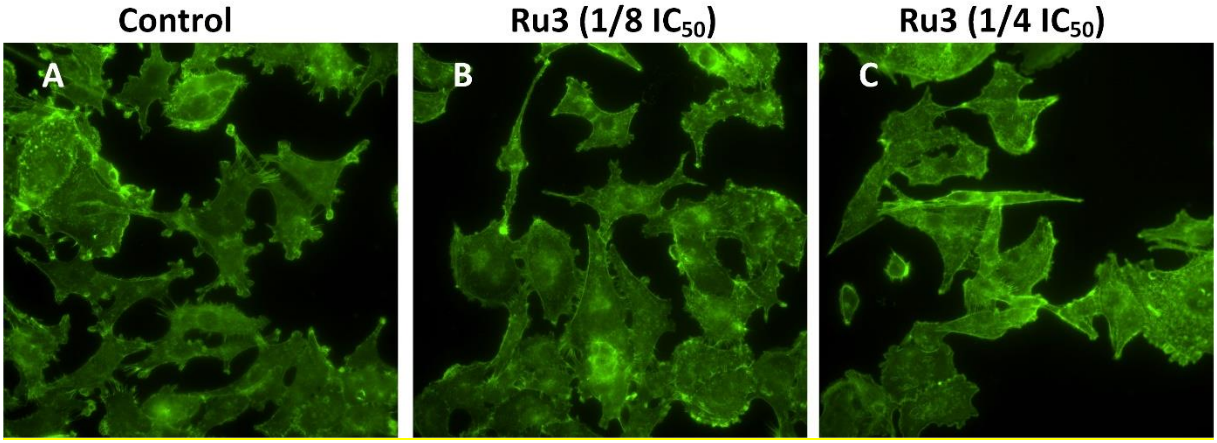

Fig. S10. Representative fluorescent images of MDA-MB-231 cells treated with different concentrations of **[Ru(dip)$_2$(bpy-SC)]Cl$_2$ (Ru3**) for 24 h (**B**, **C**). Cells treated with vehicle were used as control (**A**). The F-actin filaments were visualized using ActinGreen 488 ReadyProbes.
